# Increased fruit intake is associated with reduced risk of depression: evidence from cross-sectional and Mendelian randomization analyses

**DOI:** 10.3389/fpubh.2023.1276326

**Published:** 2023-12-13

**Authors:** Zhaoqi Yan, Yifeng Xu, Keke Li, Liangji Liu

**Affiliations:** ^1^Graduate School, Jiangxi University of Traditional Chinese Medicine, Nanchang, Jiangxi, China; ^2^Department of Respiratory and Critical Care Medicine, Hospital of Jiangxi University of Traditional Chinese Medicine, Nanchang, Jiangxi, China

**Keywords:** depression, fruit intake, the National Health and Nutrition Examination Survey, Mendelian randomization, causal relationship

## Abstract

**Background:**

The association between dietary patterns and depression has gained significant attention, but the relationship between fruit intake and depression remains unclear. This study aimed to investigate the role of fruit intake in the risk of depression using data from the National Health and Nutrition Examination Survey (NHANES) and Mendelian randomization (MR) analysis, and further explore the causal relationship between them.

**Materials and methods:**

Cross-sectional analysis was conducted using the 2005–2018 NHANES data. Specialized weighted complex survey design analysis software was used for multivariate logistic analysis. Additionally, genetic variants for fruit intake and depression, as well as its related neuroticism traits, from the GWAS were used as instrumental variables in MR analysis. The inverse variance weighted (IVW) method was employed as the primary analysis method to evaluate the causal relationship between them. MR-Egger regression, MR-PRESSO test, and leave-one-out analysis were conducted to assess heterogeneity and pleiotropy.

**Results:**

In NHANES, compared to the lowest quartile (Q1, <0.12 cup], the highest quartile (Q4, >1.49 cups) of fruit intake showed a significant reduction in the risk of depression after adjusting for relevant covariates. Model 3, after rigorous adjustment for multiple covariates, demonstrated improved predictive performance in both Receiver operating characteristic (ROC) curve and Decision curve analysis (DCA). In Model 3, the proportion of reduced depression risk associated with fruit intake reached 31% (OR = 0.69, 95% CI: 0.50–0.95). This association remained significant in the MR analysis (OR = 0.92, 95% CI = 0.87–0.96; *p* = 5.09E-04). Fruit intake was also associated with a decreased risk of neuroticism traits related to depression, including feeling lonely (OR = 0.82, 95% CI = 0.74–0.90; *p* = 2.91E-05), feeling miserable (OR = 0.79, 95% CI = 0.72–0.87; *p* = 2.35E-06), feeling fed-up (OR = 0.75, 95% CI = 0.68–0.83; *p* = 2.78E-08), irritable mood (OR = 0.89, 95% CI = 0.79–0.99; *p* = 0.03), and neuroticism (OR = 0.85, 95% CI = 0.76–0.96; *p* = 9.94E-03). The causal relationship between feeling lonely and fruit intake was bidirectional.

**Conclusion:**

Increased fruit intake has a causal effect in reducing the risk of depression and is beneficial for related psychological well-being.

## Introduction

Approximately one-fourth of the global population experiences a mental disorder at some point in their lives, with depression being a common condition. In fact, depression is now the third leading cause of disability worldwide (1). The causes of depression are complex, with reduced neurogenesis, chronic inflammation, and increased oxidative stress being potential mechanisms. In recent years, the association between diet and depression has received widespread attention, with studies suggesting that a Mediterranean diet reduces the risk of depression (2), while a Western diet (high in sugar and fat) increases the risk (3). Fruits, due to their anti-inflammatory and antioxidant properties, are considered beacons in the prevention of various diseases. As early as 2015, the US Dietary Guidelines Advisory Committee (DGAC) report highlighted the importance of fruit intake in preventing certain chronic diseases (4). However, little is known about the effects of fruit intake on mental disorders. Additionally, assessing the role of a single element in a specific dietary pattern is unreliable due to the synergistic effects of different foods (5). Generally, recommending the consumption of food groups is more consistent between dietary guidelines and research results than focusing on individual nutrients (6). Furthermore, studies on food groups are often a prerequisite for nutrient research. Therefore, the meta-analyses by Liu (7) and Saghafian (8), the review by Tuck (9) all aim to explore the benefits of consuming fruits and similar foods for mental health. However, there is some controversy surrounding these studies (10, 11). To date, the relationship between fruit and depression remains a novel and interesting area of research, but there is limited and challenging research available. Since the intervention involves food, it is difficult to conduct double-blind and placebo-controlled trials (12), making certain aspects of the typical randomized controlled trial (RCT) design unsuitable for exploring the long-term relationship between diet and disease.

The aim of this study is to investigate the correlation between fruit intake levels and the risk of depression using cross-sectional data from the National Health and Nutrition Examination Survey (NHANES). The NHANES study is a multi-stage, stratified, and nationally representative study of the US population conducted by the National Center for Health Statistics of the Centers for Disease Control and Prevention. It aims to assess the nutrition and health status of Americans and includes demographic, dietary, examination, laboratory, and questionnaire data. To avoid the potential for reverse causation, i.e., higher levels of mental health promoting better diets, including higher fruit intake (13), it is necessary to explore the causal relationship between fruit intake and depression. To date, there is a lack of intervention studies supporting a causal relationship between the two (14). Therefore, this study will also incorporate Mendelian randomization (MR) analysis to validate the conclusions of the NHANES cross-sectional study from a genetic variation perspective and further assess the causal relationship, which is difficult to achieve with traditional observational studies. MR is an analytical method that investigates how certain behaviors, environments, or other factors lead to specific health outcomes by utilizing human genetic variation to study the causal effects of modifiable disease exposures (15). Due to the random segregation of the two alleles of a single nucleotide polymorphism (SNP) according to Mendel’s law, MR has a natural advantage over traditional cohort study methods in terms of being less susceptible to confounding factors, which will help detect the causal effect of fruit intake on the risk of depression (16).

## Materials and methods

### Study population in NHANES

**Figure 1:**
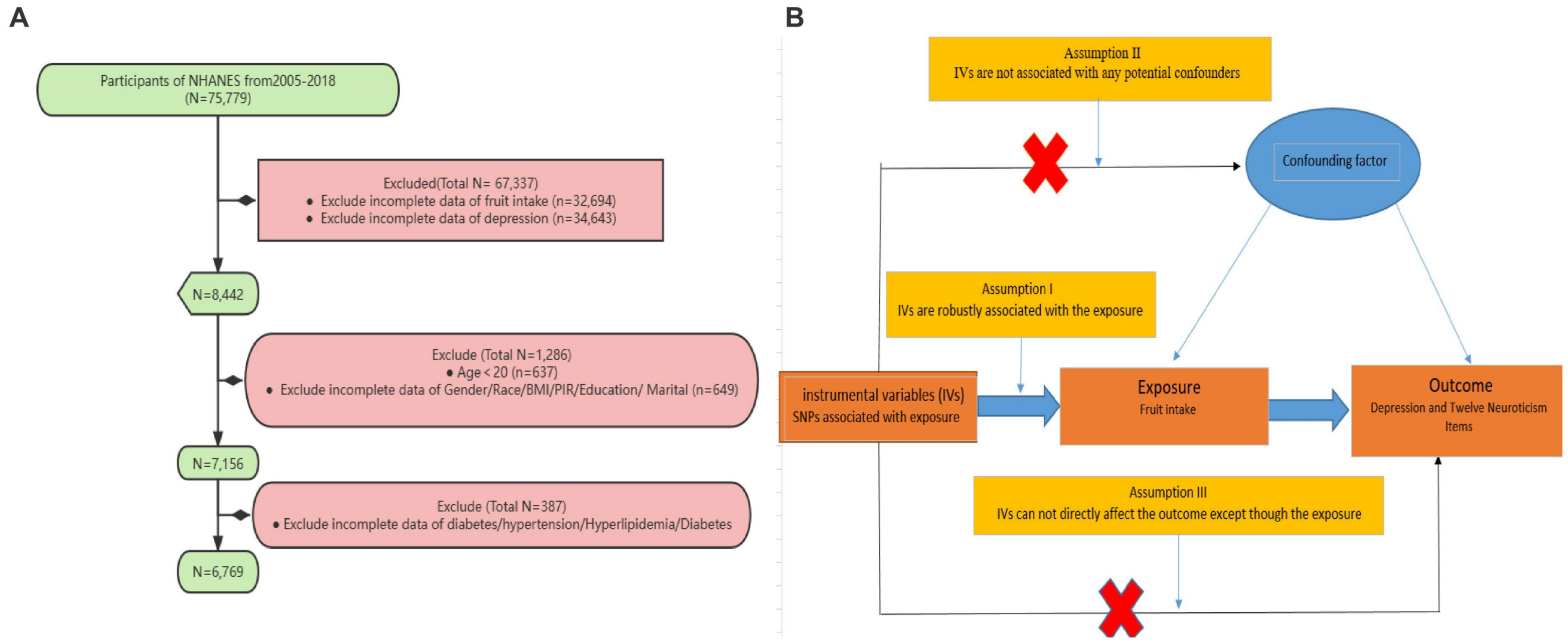
**(A)** Sampling flow during NHANES data analysis. Poverty income ratio (PIR), Body mass index (BMI) **(B)** Causal relationships between fruit intake and depression and its related neuroticism traits based on Mendelian randomization with three fundamental assumptions: (1) Hypothesis 1: Single nucleotide polymorphisms (SNPs) are strongly associated with fruit intake; (2) Hypothesis 2: The selected SNPs are not influenced by other confounding factors; (3) Hypothesis 3: SNPs affect the outcome risk by influencing the level of fruit intake, without involving any other pathways.

For this study, we analyzed data collected between 2005 and 2018 from NHANES. Subjects were excluded from the study if they had missing depression data, missing fruit intake data, were under 20 years old, or had missing covariate data (such as hypertension, hyperlipidemia, diabetes, etc.) (Figure 1A).

#### Diagnosis of depression

The diagnosis of depression was determined based on the Patient Health Questionnaire-9 (PHQ-9). The PHQ-9 is a self-administered version of the PRIME-MD diagnostic instrument for common mental disorders, with the ninth section specifically designed for depression. The PHQ-9 questionnaire consists of 9 items that assess the severity of depressive symptoms experienced by the respondent in the past 2 weeks or more. Each item is scored from “0” (not at all) to “3” (nearly every day), with a maximum score of 27. A score greater than 5 is indicative of depression (17).

#### Fruit intake

Data on fruit intake were obtained from the Food Portion and Edible Portion Database (FPED), which provides information on food portion sizes and edible portions. In this study, data on fruit intake were obtained from dietary interviews conducted as part of the FPED. Fruit intake was recorded for 2 days for each participant, and the average was taken as the daily fruit intake. Fruit intake included whole fruits, fruit slices, and fruit juice, and covered categories such as citrus fruits, melons, berries, other fruits, and fruit juice. The unit of measurement for assessing fruit intake is defined as one cup, which is equivalent to a standardized amount of fruit. The equivalent weight is determined by aggregating similar types of fruits and rounding it to the nearest 0 or 5 g. This standardized measurement method was used for food intake assessment and research purposes. For example, berries such as blackberries, blueberries, raspberries, and strawberries have weights ranging from 140 to 145 g with most weighing 145 g. Therefore, one cup of fresh or raw berries was designated as 145 g.

#### Covariates

Our covariates included age, sex, race/ethnicity (Mexican American, other Hispanic, non-Hispanic white, non-Hispanic black, non-Hispanic Asian, other race), body mass index (BMI), marital status, education level (less than 9th grade, 9–11 grade, high school graduate, some college/AA degree, and college graduate), poverty income ratio (PIR), hypertension, Hyperlipidemia, and diabetes. BMI was divided into three categories: Normal (<25 kg/m^2^), Overweight (≥25 kg/m^2^, <30 kg/m^2^), and Obese (≥30 kg/m^2^). PIR was divided into three categories: Low (≤1.39), Medium (>1.39, <=3.49), and High (>3.49). Hypertension was defined according to the American Heart Association/American College of Cardiology (AHA/ACC) 2017 guidelines as systolic blood pressure ≥ 130 mmHg or diastolic blood pressure ≥ 80 mmHg and self-reported diagnosis or use of antihypertensive medication. As per the guidelines set by the Adult Treatment Panel III (ATP 3) of the National Cholesterol Education Program (NCEP), hyperlipidemia is defined by the following criteria: total cholesterol levels equal to or exceeding 200 mg/dL, triglyceride levels equal to or exceeding 150 mg/dL, HDL cholesterol levels below 40 mg/dL for men and below 50 mg/dL for women, or LDL cholesterol levels equal to or exceeding 130 mg/dL (18). Additionally, diabetes mellitus (DM) as any of the following: (1) HbA1c levels equal to or greater than 6.5%; (2) serum glucose levels exceeding 200 mg/dL at 2 h after a 75 g glucose load (OGTT); (3) fasting glucose levels equal to or greater than 126 mg/dL; (4) self-reported diagnosis of diabetes; (5) self-reported use of insulin or other diabetes medication. The duration of diabetes was determined by subtracting the participant’s current age from the self-reported age at diagnosis, or zero for individuals diagnosed during the NHANES examination.

### NHANES analysis

Fruit intake was divided into four categories (Q1, Q2, Q3, and Q4) using quartiles, with Q1 as the reference category. Continuous variables were reported as mean ± standard deviation (SD), while categorical variables were presented as counts (N) and percentages (%). Weighted *t*-tests or weighted chi-square tests were used to assess differences between depression and non-depression subjects. Kruskal-Wallis tests or weighted chi-square tests were used to evaluate differences among the four groups based on fruit intake exposure levels. Three models were fitted: Model 1 adjusted for age, sex, and race; Model 2 further adjusted for PIR, BMI, marital status, and education level; Model 3 additionally adjusted for hyperlipidemia, hypertension, and DM. Odds ratios (OR) with 95% confidence intervals (95% CI) were calculated. Subgroup analyses were conducted for significant results. Logistic regression models were used to assess the interaction between fruit intake and covariates on depression.

In addition, Nomogram diagram were used to assess the magnitude of the risk of depression associated with covariates. To further evaluate the sensitivity of the constructed models, the performance of the models was assessed using the area under the receiver operating characteristic (ROC) curve (AUC). Decision curve analysis (DCA) was conducted to estimate the net benefit at different threshold probabilities, determining the clinical utility of the bar charts we developed (19).

### Mendelian randomization analysis

Under the framework of two-sample Mendelian randomization studies, this study utilizes SNPs as instrumental variables (IVs) for MR analysis, and adheres to the three fundamental assumptions of MR analysis. Assumption I posits that genetic variations are robustly associated with exposure (Fruit intake). To minimize bias, it is important to use IVs with strong correlations (*p* < 5 × 10^−8^), and the F-statistic of the genetic tool should be above 10. Assumption II states that any confounding factors that influence the relationship between genetic variation and the outcome of exposure are not relevant. To address this issue, we restricted the sample population to Europeans. Additionally, we obtained phenotype information for each SNP from Phenoscanner^1^ and manually excluded any SNPs that were found to have an impact on outcome-related phenotypes. Assumption III asserts that the impact of genetic variation on the outcome can only be achieved through its association with the exposure. To address this issue, we utilized MR-Egger regression and the Mendelian Randomization Pleiotropy RESidual Sum and Outlier (MR-PRESSO) method to minimize the potential for horizontal pleiotropy and conducted sensitivity analyze (Figure 1B).

#### Selection of genetic instruments

Depression is defined as seeking help for psychological health difficulties through self-reported behavior. It is diagnosed if the individual answers affirmatively to either of the following questions: “Have you ever seen a general practitioner (GP) for treatment of nerves, anxiety, tension, or depression?” or “Have you ever seen a psychiatrist for nerves, anxiety, tension, or depression?” A total of 113,769 depression patients and 208,811 healthy controls were included in the study (20). As depression manifests in various forms, it is typically driven by multiple underlying neuroticism-related factors. Therefore, data on 12 binary neuroticism items from Nagel’s study (21) were obtained. These items were derived from the revised Eysenck Personality Questionnaire and have a high genetic correlation with depression. Neuroticism is considered an important phenotype in psychiatric genetics research and can also serve as a detection tool for depression (22).

Data on fresh fruit consumption were obtained from the UK Biobank, the largest biomedical sample database (23). A total of 446,462 participants were asked the question: “About how many pieces of FRESH fruit would you eat per DAY? (Count one apple, one banana, 10 grapes etc. as one piece; put ‘0’ if you do not eat any).”

#### Statistical power

The strength of the SNPs used as instruments was assessed using the F-statistic. Only SNPs with an F-statistic greater than 10 were considered to minimize weak instrumental bias. The F-statistic was calculated using the equation: F = [R^2^ (N-K-1)/K (1- R^2^)]. (N denotes the sample size of the exposure factor, k denotes the number of SNPs in each instrument, and R^2^ denotes the variance explained by the instrument. R^2^ = 2 × EAF× (1-EAF) × Beta^2^).

#### Statistical analysis

Exposure’ IVs were selected based on a threshold of *p* < 5 × 10^−8^ and LD *r*^2^ ≤ 0.001. The primary analysis used the inverse variance weighting (IVW) method, supplemented by weighted median, weighted mode, MR-Egger, and simple mode.

#### Pleiotropy and sensitivity analysis

MR-Egger regression was used to assess the possibility of horizontal pleiotropy, with the intercept term representing the average pleiotropic effect of the IVs. MR-PRESSO was used as a supplement to detect and correct for horizontal pleiotropy by removing outliers. Cochran’s Q statistic was used to quantify heterogeneity. A leave-one-out analysis was performed to assess the influence of each peripheral SNP on the results.

The statistical analyses were conducted using R (version 4.2.2), and the MR analysis utilized the R package “TwoSampleMR.” A two-tailed *p*-value <0.05 was considered statistically significant.

## Results

### The baseline characteristics of the participants

**Figure 2:**
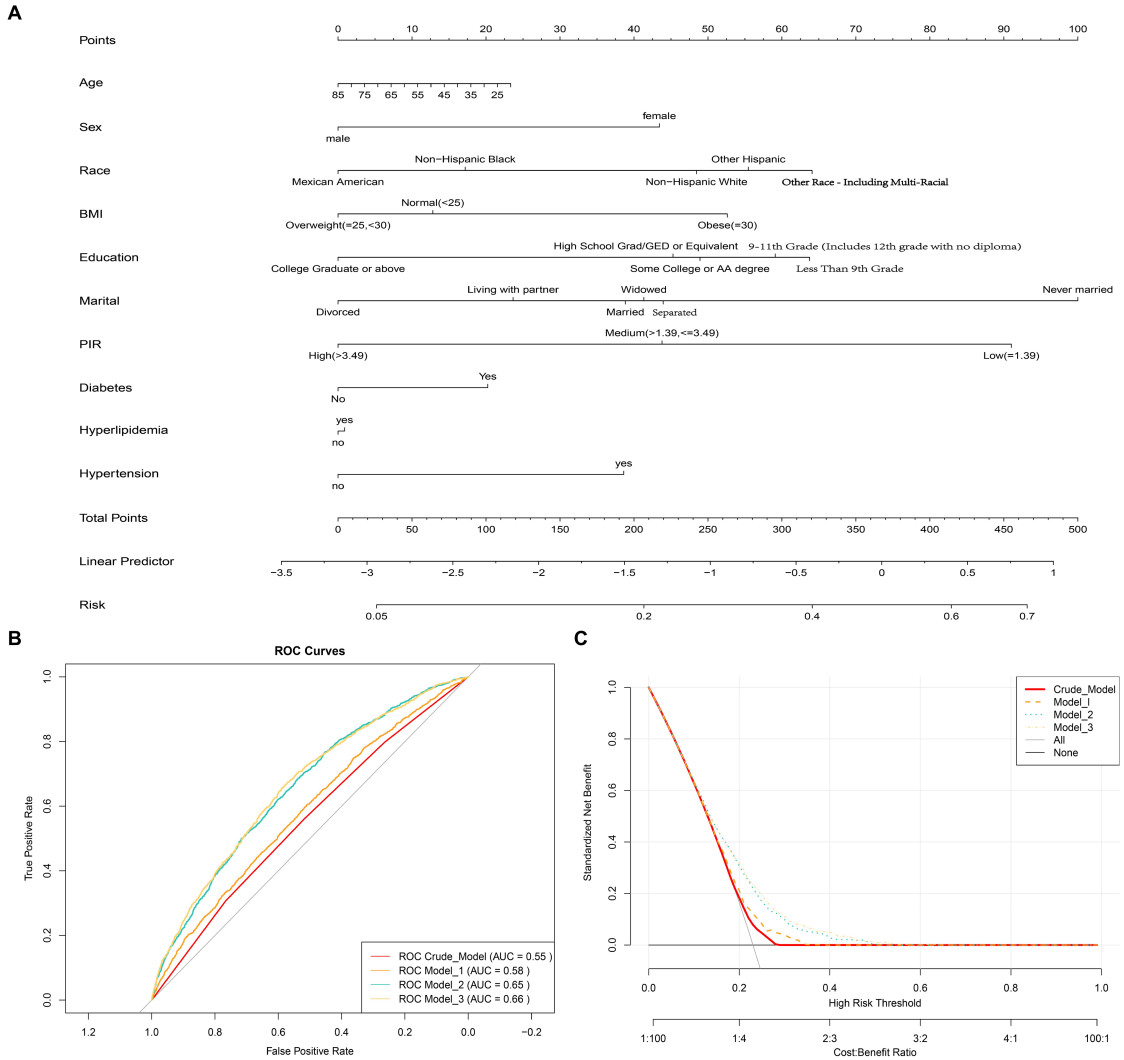
**(A)** Nomogram diagram used to predict the risk of depression, evaluating Age, Sex, Race, Education, Marital, Poverty income ratio (PIR), Body mass index (BMI), Hypertension, Hyperlipidemia, and Diabetes as risk factors. Each predictive factor has a scoring point, and the total score of these factors may indicate the risk of depression. For example, a 45-year-old female who is obese, has a low PIR level, and also suffers from hypertension and diabetes would have a depression score of 75.5 (15 + 42.5 + 52.5 + 90 + 40 + 20), corresponding to a depression risk of approximately 30%. **(B)** Receiver Operating Characteristic (ROC) curves showing the area under the curve (AUC) for the Crude model, Model 1, Model 2, and Model 3, which are 0.58, 0.55, 0.65, and 0.66, respectively. **(C)** Decision curve analysis showing the net benefit curve of the line graph model. The X-axis represents the threshold probability of depression, and the Y-axis represents the net benefit. The red, orange, sky blue, and yellow lines represent the improved predictive line graphs of the Crude model, Model 1, Model 2, and Model 3, respectively. The gray line represents the assumption that all patients use the line graph model. The black line represents the assumption that no patients use the line graph model to predict depression risk. Our study suggests that all constructed models can provide additional benefits for predicting a reduction in depression risk through fruit intake and do not produce any side effects.

A total of 6,769 individuals were included in this study. Among them, 1,559 participants were classified as having depression based on the exclusion criteria. Compared to non-depression participants (5,210 individuals), those with depression had a higher proportion of females, higher BMI, higher prevalence of hypertension and diabetes, lower proportion of individuals with higher education, and lower household income. Additionally, individuals with depression were more likely to have comorbidities of hypertension and diabetes. Importantly, it was found that individuals with depression had significantly lower fruit intake compared to non-depression participants (Table 1). Fruit intake was categorized into four quartiles using the quartile method: Q1 (<0.12 cup], Q2 (0.12–0.71 cup], Q3 (0.71–1.49 cups] and Q4 (>1. 49 cups). It was observed that the proportion of individuals with depression decreased with increasing fruit intake (Supplementary Table S1). Nomogram diagram was also used to illustrate the associations between risk factors for depression and gender, BMI, marital status, education, income, hypertension, and diabetes (Figure 2A).

**Table 1:** Characteristics of participants by depression or non- depression.

Characteristic	Overall, *N* = 6,769 (100%)^1,2^	No, *N* = 5,210 (78%)^1,2^	Yes, *N* = 1,559 (22%)^1,2^	*p* value
Age (years)	47.2 (16.7)	47.2 (16.8)	47.2 (16.6)	>0.9
Sex				**<0.001**
Female	3,586 (53%)	2,628 (51%)	958 (60%)	
Male	3,183 (47%)	2,582 (49%)	601 (40%)	
Race				0.11
Non-Hispanic White	3,072 (70%)	2,358 (71%)	714 (68%)	
Non-Hispanic Black	1,563 (11%)	1,202 (10%)	361 (12%)	
Mexican American	1,046 (7.2%)	826 (7.3%)	220 (6.7%)	
Other race - including multi-racial	703 (7.1%)	545 (6.9%)	158 (8.0%)	
Other Hispanic	385 (4.7%)	279 (4.4%)	106 (5.6%)	
Body mass index (BMI)				**<0.001**
Normal(<25)	1,882 (30%)	1,498 (30%)	384 (27%)	
Obese(≥30)	2,670 (39%)	1,919 (36%)	751 (49%)	
Overweight(≥25,<30)	2,217 (31%)	1,793 (33%)	424 (24%)	
Education				**<0.001**
9–11th Grade (Includes 12th grade with no diploma)	846 (8.5%)	599 (7.7%)	247 (11%)	
College graduate or above	1,600 (30%)	1,362 (33%)	238 (19%)	
High school grad/GED or equivalent	1,613 (25%)	1,209 (24%)	404 (29%)	
Less than 9th grade	545 (3.8%)	401 (3.6%)	144 (4.7%)	
Some college or AA degree	2,165 (32%)	1,639 (31%)	526 (35%)	
Marital				**<0.001**
Divorced	3,664 (57%)	2,968 (60%)	696 (47%)	
Living with partner	492 (5.1%)	355 (4.7%)	137 (6.8%)	
Married	724 (11%)	513 (9.9%)	211 (13%)	
Never married	221 (2.5%)	140 (1.9%)	81 (4.5%)	
Separated	1,078 (16%)	800 (16%)	278 (19%)	
Widowed	590 (8.4%)	434 (8.0%)	156 (10%)	
Poverty income ratio (PIR)				**<0.001**
High(>3.49)	2,295 (46%)	1,946 (50%)	349 (32%)	
Low(≤1.39)	1,890 (19%)	1,290 (16%)	600 (29%)	
Medium(>1.39,<=3.49)	2,584 (35%)	1,974 (34%)	610 (39%)	
Hypertension	2,857 (37%)	2,108 (35%)	749 (44%)	**<0.001**
Hyperlipidemia	4,658 (67%)	3,551 (67%)	1,107 (70%)	0.2
Diabetes	1,233 (14%)	887 (13%)	346 (18%)	**0.002**
Fruit-total	0.94 (1.10)	0.98 (1.12)	0.80 (1.00)	**<0.001**

(NHANES 2005–2018, *N* = 6,769).

^1^Mean ± SD for continuous; *n* (%) for categorical.

^2^*t*-test adapted to complex survey samples; chi-squared test with Rao & Scott’s second-order correction.

Values in bold represent statistically significant differences (*p* < 0.05).

### Associations between fruit intake and depression outcomes

Using multiple logistic regression models, it was found that in the Crude Model, all three quartile groups (Q2, Q3, and Q4) were significantly negatively associated with the risk of depression compared to the reference group (Q1) (*p* < 0.05). After adjusting for multiple covariates (age, sex, race, education, marital status, PIR, hypertension, hyperlipidemia, and diabetes), only the Q4 group remained robustly associated with a lower risk of depression (Table 2). This suggests that when daily fruit intake exceeds 1.49 cups, the average risk of depression decreases by 31%. Interaction analysis was conducted to examine the potential effect modification of fruit intake by gender, education, BMI, PIR, hypertension, and diabetes, which were significant covariates associated with depression and non-depression (P for interaction >0.05, Table 2). This further confirms the robustness of the findings. The ROC curve showed an AUC of 0.66 for Model 3, indicating good predictive ability (Figure 2B). The DCA demonstrated that Model 3 consistently had a net benefit probability of 0 to 50%, which was always higher than the assumption of no intervention (represented by the black solid line None group) (Figure 2C). These results indicate that all constructed models can provide additional benefits for predicting the risk of depression reduction in fruit intake without any side effects.

**Table 2:** Weighted multivariate adjusted logistic regression and subgroup analysis of depression risk with different fruit intake levels in NHANES from 2005 to 2018.

Regression model	Crude model OR (95% CI)	Model 1 OR (95% CI)	Model 2 OR (95% CI)	Model 3 OR (95% CI)
Fruit-total (cup)				
Q1(<0.12]	Reference	Reference	Reference	Reference
Q2(0.12–0.71]	0.77 (0.60, 0.98) *	0.72 (0.56, 0.91) **	0.83 (0.62, 1.11)	0.83 (0.60, 1.14)
Q3(0.71–1.49]	0.70 (0.57, 0.87) **	0.67 (0.54, 0.83) **	0.75 (0.57, 0.98) *	0.75 (0.56, 1.00)
Q4 (>1.49)	0.58 (0.47, 0.73) ***	0.56 (0.44, 0.71) ***	0.69 (0.52, 0.91) *	0.69 (0.50, 0.95) *

Subgroup	Fruit-total-Q2(0.12–0.71] OR(95%CI)	Fruit-total-Q3 (0.71–1.49] OR(95%CI)	Fruit-total-Q4(>1.49) OR(95%CI)	Interaction *p* value
Sex				*p* = 0.47
Female	0.70(0.44, 1.12)	0.69(0.48, 0.99) *	0.62(0.42, 0.90) *	
Male	1.04(0.70, 1.56)	0.79(0.56, 1.13)	0.75(0.51, 1.11)	
Education				*p* = 0.45
9–11th grade (Includes 12th grade with no diploma)	0.91(0.56, 1.48)	0.79(0.45, 1.40)	1.22(0.53, 2.82)	
College graduate or above	0.81(0.30, 2.20)	0.93(0.42, 2.09)	0.56(0.21, 1.48)	
High school grad/GED or equivalent	0.78(0.47, 1.32)	0.65(0.42, 1.01)	0.82(0.43, 1.56)	
Less than 9th grade	1.66(0.59, 4.69)	0.98(0.33, 2.90)	1.67(0.68, 4.11)	
Some college or AA degree	0.85(0.59, 1.22)	0.74(0.49, 1.11)	0.60(0.36, 1.00)	
BMI (Kg/m^2^)				*p* = 0.46
Normal(<25)	0.96(0.73, 1.28)	0.92(0.66, 1.29)	0.75(0.53, 1.06)	
Obese(≥30)	0.83(0.53, 1.30)	0.66(0.38, 1.15)	0.60(0.36, 1.00)	
Overweight(≥25,<30)	0.68(0.34, 1.36)	0.64(0.35, 1.16)	0.69(0.40, 1.19)	
PIR				*p* = 0.48
High(>3.49)	0.88(0.46, 1.68)	0.71(0.40, 1.26)	0.64(0.31, 1.31)	
Low(≤1.39)	0.91(0.63, 1.31)	0.97(0.67, 1.39)	0.81(0.56, 1.17)	
Medium(>1.39,<=3.49)	0.73(0.47, 1.13)	0.69(0.47, 1.00)	0.66(0.44, 1.00)	
Hypertension				*p* = 0.43
Yes	0.88(0.59, 1.32)	0.68(0.48, 0.96)*	0.62(0.44, 0.87)*	
No	0.71(0.50, 1.02)	0.81(0.55, 1.20)	0.78(0.51, 1.18)	
Diabetes				*p* = 0.48
Yes	0.83(0.59, 1.16)	0.70(0.51, 0.96)*	0.65(0.47, 0.90)*	
No	0.86(0.48, 1.55)	1.05(0.66, 1.66)	1.00(0.48, 2.07)	

*p < 0.05; **p < 0.01; ***p < 0.001.

Multiple logistic regression model: Model 1: Adjusted for Age, Sex, Race, Model 2: Adjusted for Age, Sex, Race, Education, Marital, Poverty income ratio (PIR), Body mass index (BMI), Model 3: Adjusted for Age, Sex, Race, Education, Marital, PIR, BMI, Hypertension, Hyperlipidemia, Diabetes.

Subgroup Analysis Adjustment Factors: Age; Sex; Race; Education; Marital; PIR; BMI; Hypertension; Hyperlipidemia; Diabetes, excluding sub-group variables, and the reference object in the sub-group is Q1 of Fruit-total intake level.

### MR estimates

**Figure 3:**
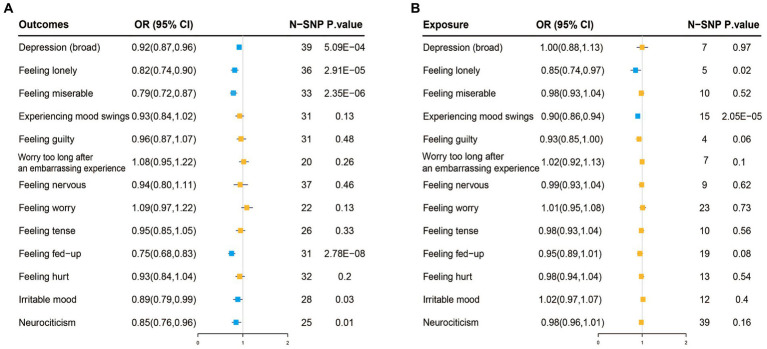
**(A)** Two-sample Mendelian randomization analysis with inverse-variance weighted (IVW) as the primary method, where exposure is fruit intake and the outcome is depression and its 12 related neuroticism traits. The blue color represents causal associations where exposure has a protective effect on the outcome, while the orange color represents no causal association between exposure and the outcome. **(B)** Reverse Mendelian randomization analysis with IVW as the primary method, where exposure is depression and its 12 related neuroticism traits, and the outcome is fruit intake. The blue color represents causal associations where exposure has a protective effect on the outcome, while the orange color represents no causal association between exposure and the outcome.

When fruit intake was used as an exposure instrument and depression as an outcome instrument, the genetic instrument strength, indicated by a Total-F-statistic of 46.3 (Supplementary Table S2), suggested no statistical bias due to weak instrument bias. In the IVW analysis, a significant inverse association between fruit intake and the risk of depression was observed (OR = 0.92, 95% CI = 0.87–0.96; *p* = 5.09E-04). All alternative analysis methods yielded directional amplitudes consistent with the IVW analysis. No evidence of heterogeneity or horizontal pleiotropy was observed, as indicated by non-significant Cochran’s Q test and MR-Egger intercept/MR-Presso *p*-values >0.05 (Table 3). Leave-one-out analysis demonstrated the stability of the results, showing consistent findings even when individual SNPs were excluded. These findings provide robust statistical evidence supporting the inverse association between fruit intake and the risk of depression. Additionally, symptoms related to depression such as feeling lonely, feeling miserable, feeling fed-up, irritable mood, and neuroticism were also inversely associated with fruit intake (Figure 3A; Supplementary Table S3). Sensitivity analysis showed heterogeneity in feeling nervous but no horizontal pleiotropy (Table 3). The stability of the results was also confirmed in the leave-one-out analysis (Supplementary Figures S1–S4).

**Table 3:** Causal relationship and sensitivity analysis of fruit intake and depression and its related neuroticism traits in a two-sample Mendelian randomization analysis.

Exposure	Outcome	Cochran Q- *p*. value	MR-Egger intercept-*p*. value	MR-PRESSO- *p*. value
Fruit intake	Depression	0.10	0.58	0.12
	Feeling nervous	7.49E-11	0.94	0.05
	Feeling worry	0.64	0.72	0.63
	Irritable mood	0.11	0.53	0.12
	Feeling lonely	0.52	0.10	0.12
	Feeling tense	0.41	0.33	0.30
	Feeling guilty	0.24	0.95	0.10
	Feeling hurt	0.17	0.63	0.18
	Feeling fed-up	0.46	0.34	0.15
	Neurociticism	0.31	0.34	0.35
	Feeling miserable	0.68	0.98	0.29
	Worry too long after an embarrassing experience	0.99	0.93	0.99
	Experiencing mood swings	0.53	0.11	0.29

The MR-Egger intercept and MR-PRESSO *p*-values in the table are used to investigate the presence of horizontal pleiotropy. A *p*-value > 0.05 indicates the absence of horizontal pleiotropy, suggesting that the study aligns with the basic assumptions of Mendelian randomization. On the other hand, the *p*-value of Cochran’s Q test explores the presence of heterogeneity. A *p*-value > 0.05 indicates no significant heterogeneity, indicating an association between the instrumental variables and phenotype.

Reverse MR analysis showed no association between depression and fruit intake. However, feeling lonely (IVW, OR: 0.85, 95% CI: 0.74–0.97, *p* = 0.02) and mood swings (IVW, OR: 0.9, 95% CI: 0.86–0.94, *p* = 2.05E-05) among the twelve neuroticism traits were inversely associated with fruit intake (Figure 3B; Supplementary Table S4). Sensitivity analysis also confirmed the reliability of the results (Supplementary Table S5; Supplementary Figures S4–S7).

## Discussion

This study is the first to investigate the impact of fruit intake on depression from a cross-sectional and genetic perspective, and it is also the first to apply MR analysis to examine the association between fruit intake and depression. The cross-sectional results from NHANES support a strong correlation between higher levels of fruit intake (>1.49 cups) and a reduced risk of depression, which has been validated by ROC and DCA. Meanwhile, the MR analysis indicates only a positive causal association between fruit intake and depression. For the 12 neuroticism items related to depression, there is a positive causal association between fruit intake and loneliness, miserableness. Fed-upness, irritability, and neuroticism, with bidirectional causality observed for loneliness.

Previous systematic reviews have also indicated the benefits of fruit consumption on various aspects of mental health (24), including some neuroticism items (loneliness, miserableness, fed-upness, irritability, and neuroticism) that are causally associated with fruit consumption. These symptoms are common in depression, further confirming that fruit consumption can prevent a wide range of depressive states, which are often precursors to major depression. This provides us with some insights, suggesting that individuals at risk of depression due to family history, dysfunctional cognitions, stressful life events and circumstances, and interpersonal relationship difficulties should be recommended to consume more than 1.49 cups of fruit daily. For example, daily consumption of 210.25 g of fresh berries, 268.25 g of citrus fruit or oranges, and 362.5 g of 100% fresh fruit juice (recommended fruit intake amounts are shown in Supplementary Table S6).

The subgroup analysis of this study suggests that fruit consumption has a more significant effect on female depression patients compared to males. Females are more susceptible to the effects of hormonal changes in the menstrual cycle on neurotransmitters in the brain, and postpartum depression is also a significant concern (25). Additionally, the social roles that females play, such as career, family, and social interactions, may increase their risk of depression due to the pressures and expectations associated with these roles. Furthermore, females generally have poorer psychological resilience (26). Hypertension and diabetes are often accompanied by increased rates of complications and rising healthcare costs, which significantly reduce quality of life for patients and lead to heavy economic and psychological burdens. As a result, the individual’s happiness index will decrease significantly over time, leading to the development of depression (27, 28). Therefore, these adults often have comorbid depression. However, the differences observed in the subgroup analyses did not have a decisive impact on the relationship between fruit intake and depression (interaction *p*-values were all greater than 0.05, Table 2), but they only suggest that populations with these factors may need to pay more attention to our conclusions.

Fruits are a valuable source of dietary nutrition, rich in complex carbohydrates, various vitamins, and polyphenolic antioxidants (29). It is known that some nutritional imbalances can increase the risk of depression, including but not limited to macronutrients (dietary sugar, fat, and protein) and micronutrients (polyphenol, minerals and vitamins). As an illustration, a deficiency in any B vitamin or folate can lead to homocysteine accumulation and harmful cellular effects, which negatively affect the central nervous system (30). Vitamins C, E, and D play important roles in endothelial cells in signaling cascades and are also crucial for regulating neurotrophic factors, neuroprotection, neuroplasticity, brain development, and neuroimmune regulation (31, 32). Fruits rich in polyphenolic compounds, such as berries, have been substantiated through various randomized controlled trials to effectively reduce the risk of depression (33, 34). This is attributed to their antioxidative and anti-inflammatory properties (35), as well as their ability to elevate BDNF (brain-derived neurotrophic factor) levels (36). Trace metals abundant in fruits also have profound significance for the nervous system, similar to antioxidants (such as vitamins and polyphenols). For example, magnesium not only participates in maintaining the flow of neurons in the brain and various biological processes but also reduces the concentration of plasma C-reactive protein (37). Iron and zinc deficiencies induce neurological and physical symptoms and psychiatric symptoms related to depression (38), which may be related to BDNF and oxidative stress levels (39). Zinc supplementation is also a treatment option for alleviating symptoms related to depression (38). Future research should delve deeper into exploring the differences among various types of fruits in reducing the risk of depression. For example, fruits with high polyphenol content, represented by berries, fruits rich in vitamins, exemplified by oranges, and fruits with high sugar and water content, symbolized by melons, warrant further investigation.

In conclusion, the possible regulatory mechanisms of fruits in depression may involve neural signal transduction, chronic inflammation, and oxidative stress. The reason why consuming fruits has a good effect is that naturally occurring trace elements are more beneficial to health. For instance, folate consumed from fortification and dietary supplements has been shown to be unrelated to depression, while folate in natural foods can reduce the risk of depression (40). Currently, there is no research on the relationship between fruit intake and individuals with a sense of loneliness, but a small amount of research has confirmed that a sense of loneliness can lead to eating disorders, including anorexia nervosa and binge eating disorder (41). A sense of loneliness and emotional fluctuations may be due to changes in neurotransmitters and neuropeptides in the body, which may further affect individual eating habits and genetic variations related to fruit intake. Such as dopamine, which is involved in controlling movement, cognition, emotion, neuroendocrine function, and reward systems. Specifically, dopamine is closely related to the food reward properties in the mesolimbic system and drives us to seek food (13). The secretion of endorphins is also related to social interaction, and a lack of endorphins can lead to food deprivation (42). These mechanisms, such as dopamine and endorphin regulation, may be potential explanations for the bidirectional causal association between loneliness and fruit intake observed in this study.

However, this study has some limitations. Firstly, fruit intake was assessed using questionnaires, and a few days of fruit intake may not represent an individual’s long-term intake level. Secondly, for individuals with depression, there may be cognitive impairments that result in poor recall of actual food consumption. However, due to our large sample size, we were able to minimize the impact of these issues. Furthermore, in the cross-sectional study of NHANES, fruit intake was measured in a specific unit (cup), while the UKB study assessed fruit intake in “pieces,” which may lead to some differences in the specific values of the degree of risk reduction for depression. This needs to be further investigated in future studies. Finally, considering that NHANES and MR data mainly come from participants of non-Hispanic and European ancestry, it is still unclear whether the same results apply to other racial/ethnic groups. However, Table 1 shows that there is no statistically significant difference in race between the depression and non-depression groups (*p* = 0.11), and race was also adjusted for as a covariate in the study, yielding robust results. This indicates that race/ethnicity does not affect the conclusions of this study.

## Conclusion

The daily consumption of fruits (>1.49 cups) may contribute to a reduced risk of depression and help alleviate psychological characteristics such as loneliness, miserableness, mood swings, irritability, and neuroticism. These conclusions demonstrate a positive causal relationship, with loneliness also showing a bidirectional causal relationship with fruit intake. Although our study results support existing public health guidelines that encourage fruit consumption as part of a healthy diet to reduce the risk of depression, further research is needed to validate our findings and explore potential underlying mechanisms.

## Data availability statement

The original contributions presented in the study are included in the article/Supplementary material, further inquiries can be directed to the corresponding authors.

## Ethics statement

The studies involving human participants were reviewed and approved by the NCHS Research Ethics Review Board CDC NCHS National Health and Nutrition Examination Survey About NHANES NCHS Ethics Review Board (ERB). Written informed consent for participation was not required in accordance with the national legislation and the institutional requirements.

## Author contributions

ZY: Data curation, Methodology, Software, Writing – original draft. YX: Conceptualization, Methodology, Writing – original draft. KL: Software, Writing – original draft. LL: Writing – review & editing.

## Footnotes

^1^ http://www.phenoscanner.medschl.cam.ac.uk/

## References

[1] Park LT Zarate CA Jr. Depression in the primary care setting. N Engl J Med. (2019) 380:559–68. doi: 10.1056/NEJMcp1712493, PMID: 30726688 PMC6727965

[2] Gibson-Smith D Bot M Brouwer IA Visser M Giltay EJ Penninx B. Association of food groups with depression and anxiety disorders. Eur J Nutr. (2020) 59:767–78. doi: 10.1007/s00394-019-01943-4, PMID: 30945032 PMC7058560

[3] Li Y Lv MR Wei YJ Sun L Zhang JX Zhang HG. Dietary patterns and depression risk: a meta-analysis. Psychiatry Res. (2017) 253:373–82. doi: 10.1016/j.psychres.2017.04.020 28431261

[4] Millen BE Abrams S Adams-Campbell L Anderson CA Brenna JT Campbell WW. The 2015 dietary guidelines advisory committee scientific report: development and major conclusions. Adv Nutr. (2016) 7:438–44. doi: 10.3945/an.116.012120 27184271 PMC4863277

[5] Tapsell LC Neale EP Satija A Hu FB. Foods, nutrients, and dietary patterns: interconnections and implications for dietary guidelines. Adv Nutr. (2016) 7:445–54. doi: 10.3945/an.115.011718, PMID: 27184272 PMC4863273

[6] Jacobs DR Tapsell LC. Food synergy: the key to a healthy diet. Proc Nutr Soc. (2013) 72:200–6. doi: 10.1017/S0029665112003011, PMID: 23312372

[7] Liu X Yan Y Li F Zhang D. Fruit and vegetable consumption and the risk of depression: a meta-analysis. Nutrition. (2016) 32:296–302. doi: 10.1016/j.nut.2015.09.009, PMID: 26691768

[8] Saghafian F Malmir H Saneei P Milajerdi A Larijani B Esmaillzadeh A. Fruit and vegetable consumption and risk of depression: accumulative evidence from an updated systematic review and meta-analysis of epidemiological studies. Br J Nutr. (2018) 119:1087–101. doi: 10.1017/S0007114518000697 29759102

[9] Tuck NJ Farrow C Thomas JM. Assessing the effects of vegetable consumption on the psychological health of healthy adults: a systematic review of prospective research. Am J Clin Nutr. (2019) 110:196–211. doi: 10.1093/ajcn/nqz080, PMID: 31152539

[10] Saneei P Saghafian F Esmaillzadeh AR. Fruit and vegetable consumption and the risk of depression: a meta-analysis. Nutrition. (2016) 32:1162–3. doi: 10.1016/j.nut.2016.01.001 27106394

[11] Kawada TR. Fruit and vegetable consumption and the risk of depression: a meta-analysis. Nutrition. (2018) 45:147. doi: 10.1016/j.nut.2017.05.013 28697855

[12] Jacobs DR Tapsell LC Temple NJ. Food synergy: the key to balancing the nutrition research effort. Public Health Rev. (2011) 33:507–29. doi: 10.1007/BF03391648

[13] Bremner JD Moazzami K Wittbrodt MT Nye JA Lima BB Gillespie CF. Diet, stress and mental health. Nutrients. (2020) 12:2428. doi: 10.3390/nu12082428, PMID: 32823562 PMC7468813

[14] Kingsbury M Dupuis G Jacka F Roy-Gagnon MH McMartin SE Colman I. Associations between fruit and vegetable consumption and depressive symptoms: evidence from a national Canadian longitudinal survey. J Epidemiol Commun Health. (2016) 70:155–61. doi: 10.1136/jech-2015-205858 26311898

[15] Burgess S Butterworth A Malarstig A Thompson SG. Use of Mendelian randomisation to assess potential benefit of clinical intervention. BMJ. (2012) 345:e7325. doi: 10.1136/bmj.e7325, PMID: 23131671

[16] Davies NM Holmes MV Davey Smith G. Reading Mendelian randomisation studies: a guide, glossary, and checklist for clinicians. BMJ. (2018) 362:k601. doi: 10.1136/bmj.k601, PMID: 30002074 PMC6041728

[17] Kroenke K Spitzer RL Williams JB. The PHQ-9: validity of a brief depression severity measure. J Gen Intern Med. (2001) 16:606–13. doi: 10.1046/j.1525-1497.2001.016009606.x, PMID: 11556941 PMC1495268

[18] National Cholesterol Education Program (NCEP) Expert Panel on Detection, Evaluation, and Treatment of High Blood Cholesterol in Adults (Adult Treatment Panel III). Third report of the National Cholesterol Education Program (NCEP) expert panel on detection, evaluation, and treatment of high blood cholesterol in adults (adult treatment panel III) final report. Circulation. (2002) 106:3143–421. doi: 10.1161/circ.106.25.3143 12485966

[19] Vickers AJ Elkin EB. Decision curve analysis: a novel method for evaluating prediction models. Med Decis Mak. (2006) 26:565–74. doi: 10.1177/0272989X06295361, PMID: 17099194 PMC2577036

[20] Howard DM Adams MJ Shirali M Clarke TK Marioni RE Davies G. Genome-wide association study of depression phenotypes in UK biobank identifies variants in excitatory synaptic pathways. Nat Commun. (2018) 9:1470. doi: 10.1038/s41467-018-03819-3 29662059 PMC5902628

[21] Nagel M Watanabe K Stringer S Posthuma D van der Sluis S. Item-level analyses reveal genetic heterogeneity in neuroticism. Nat Commun. (2018) 9:905. doi: 10.1038/s41467-018-03242-8, PMID: 29500382 PMC5834468

[22] Kendler KS Gatz M Gardner CO Pedersen NL. Personality and major depression: a Swedish longitudinal, population-based twin study. Arch Gen Psychiatry. (2006) 63:1113–20. doi: 10.1001/archpsyc.63.10.1113 17015813

[23] Higbee DH Granell R Sanderson E Davey Smith G Dodd JW. Lung function and cardiovascular disease: a two-sample Mendelian randomisation study. Eur Respir J. (2021) 58:2003196. doi: 10.1183/13993003.03196-2020, PMID: 33574079

[24] Glabska D Guzek D Groele B Gutkowska K. Fruit and vegetable intake and mental health in adults: a systematic review. Nutrients. (2020) 12:115. doi: 10.3390/nu12010115 31906271 PMC7019743

[25] Dowlati Y Ravindran AV Segal ZV Stewart DE Steiner M Meyer JH. Selective dietary supplementation in early postpartum is associated with high resilience against depressed mood. Proc Natl Acad Sci U S A. (2017) 114:3509–14. doi: 10.1073/pnas.1611965114, PMID: 28289215 PMC5380083

[26] Hammen C. Risk factors for depression: an autobiographical review. Annu Rev Clin Psychol. (2018) 14:1–28. doi: 10.1146/annurev-clinpsy-050817-084811, PMID: 29328780

[27] Ohira T. Psychological distress and cardiovascular disease: the circulatory risk in communities study (CIRCS). J Epidemiol. (2010) 20:185–91. doi: 10.2188/jea.je20100011, PMID: 20431233 PMC3900839

[28] Egede LE Ellis C. Diabetes and depression: global perspectives. Diabetes Res Clin Pract. (2010) 87:302–12. doi: 10.1016/j.diabres.2010.01.024 20181405

[29] Liu RH. Health-promoting components of fruits and vegetables in the diet. Adv Nutr. (2013) 4:384S–92S. doi: 10.3945/an.112.003517, PMID: 23674808 PMC3650511

[30] Folstein M Liu T Peter I Buell J Arsenault L Scott T. The homocysteine hypothesis of depression. Am J Psychiatry. (2007) 164:861–7. doi: 10.1176/ajp.2007.164.6.861 17541043

[31] Ma J Li K. Negative association between serum vitamin D levels and depression in a Young adult US population: a cross-sectional study of NHANES 2007-2018. Nutrients. (2023) 15:2947. doi: 10.3390/nu15132947, PMID: 37447273 PMC10346331

[32] Khosravi M Sotoudeh G Amini M Raisi F Mansoori A Hosseinzadeh M. The relationship between dietary patterns and depression mediated by serum levels of folate and vitamin B12. BMC Psychiatry. (2020) 20:63. doi: 10.1186/s12888-020-2455-2, PMID: 32054533 PMC7020545

[33] Khalid S Barfoot KL May G Lamport DJ Reynolds SA Williams CM. Effects of acute blueberry flavonoids on mood in children and Young adults. Nutrients. (2017) 9:158. doi: 10.3390/nu9020158, PMID: 28230732 PMC5331589

[34] Fisk J Khalid S Reynolds SA Williams CM. Effect of 4 weeks daily wild blueberry supplementation on symptoms of depression in adolescents. Br J Nutr. (2020) 124:181–8. doi: 10.1017/S0007114520000926, PMID: 32151287

[35] Kontogianni MD Vijayakumar A Rooney C Noad RL Appleton KM McCarthy D. A high polyphenol diet improves psychological well-being: the polyphenol intervention trial (PPhIT). Nutrients. (2020) 12:2445. doi: 10.3390/nu12082445, PMID: 32823886 PMC7469043

[36] Caracci F Harary J Simkovic S Pasinetti GM. Grape-derived polyphenols ameliorate stress-induced depression by regulating synaptic plasticity. J Agric Food Chem. (2020) 68:1808–15. doi: 10.1021/acs.jafc.9b01970 31532659

[37] Botturi A Ciappolino V Delvecchio G Boscutti A Viscardi B Brambilla P. The role and the effect of magnesium in mental disorders: a systematic review. Nutrients. (2020) 12:1661. doi: 10.3390/nu12061661, PMID: 32503201 PMC7352515

[38] Li Z Li B Song X Zhang D. Dietary zinc and iron intake and risk of depression: a meta-analysis. Psychiatry Res. (2017) 251:41–7. doi: 10.1016/j.psychres.2017.02.006, PMID: 28189077

[39] Bjorkholm C Monteggia LM. BDNF - a key transducer of antidepressant effects. Neuropharmacology. (2016) 102:72–9. doi: 10.1016/j.neuropharm.2015.10.034 26519901 PMC4763983

[40] Payne ME Jamerson BD Potocky CF Ashley-Koch AE Speer MC Steffens DC. Natural food folate and late-life depression. J Nutr Elder. (2009) 28:348–58. doi: 10.1080/01639360903417181, PMID: 21184377 PMC3324853

[41] Levine MP. Loneliness and eating disorders. J Psychol. (2012) 146:243–57. doi: 10.1080/00223980.2011.606435 22303623

[42] Tolentino L Iqbal A Rahman S Lutfy K. The role of Beta-endorphin in food deprivation-mediated increases in food intake and binge-eating. Brain Sci. (2023) 13:212. doi: 10.3390/brainsci13020212, PMID: 36831755 PMC9954518

