# Supplementary material for: Increased fruit intake is associated with reduced risk of depression: evidence from cross-sectional and Mendelian randomization analyses
Source: Front Public Health. 2023 Dec 13;11:1276326. doi: 10.3389/fpubh.2023.1276326 (PMC10753833; doi:10.3389/fpubh.2023.1276326)
Supplement: Supplementary file 1 [file Data_Sheet_1.docx]

Supplementary material content:

[Table S1. Participant characteristics by fruit intake quartile interval. (NHANES 2005-2018, N = 6,769) 2](#_Toc142609878)

[Table S2. Summary of Genome-Wide Association Studies involved in our study. 5](#_Toc142609879)

[Table S3. Causal Association between Fruit Intake and Depression and its Related Neuroticism Traits: Results of Two-Sample Mendelian Randomization Analysis. 6](#_Toc142609880)

[Table S4. Causal Association between Depression and its Related Neuroticism Traits and Fruit Intake: Results of Reverse Mendelian Randomization Analysis. 8](#_Toc142609881)

[Figure S1. Visualized leave-one-out result graph. 11](#_Toc142609882)

[Figure S2. Visualized leave-one-out result graph. 12](#_Toc142609883)

[Figure S3. Visualized leave-one-out result graph. 13](#_Toc142609884)

[Figure S4. Visualized leave-one-out result graph. 14](#_Toc142609885)

[Table S5. Causal association and sensitivity analysis between depression and fruit intake in Reverse Mendelian Randomization analysis. 15](#_Toc142609886)

[Figure S5. Visualized leave-one-out result graph. 16](#_Toc142609887)

[Figure S6. Visualized leave-one-out result graph. 17](#_Toc142609888)

[Figure S7. Visualized leave-one-out result graph. 18](#_Toc142609889)

[Table S6. Specific Values of Daily Fruit Intake (>1.45 cups) across Various Fruit Types Based on FPED Fruit Nutrient Database. 19](#_Toc142609890)

# Table S1. Participant characteristics by fruit intake quartile interval. (NHANES 2005-2018, N = 6,769)

| **Characteristic** | **Overall**, N = 6769 (100%)^1^ | **Q1**, N = 1695 (27%)^1^ | **Q2**, N = 1691 (26%)^1^ | **Q3**, N = 1693 (24%)^1^ | **Q4**, N = 1690 (22%)^1^ | **P Value**^2^ |
| --- | --- | --- | --- | --- | --- | --- |
| **Age (years)** | 47.2 (16.7) | 42.9 (15.2) | 47.4 (16.4) | 49.0 (17.1) | 50.4 (17.4) |  |
| **Sex** |  |  |  |  |  | **<0.001** |
| *Female* | 3,586 (53%) | 792 (47%) | 982 (60%) | 923 (55%) | 889 (49%) |  |
| *Male* | 3,183 (47%) | 903 (53%) | 709 (40%) | 770 (45%) | 801 (51%) |  |
| **Race** |  |  |  |  |  | **0.036** |
| *Non-Hispanic White* | 3,072 (70%) | 819 (73%) | 765 (70%) | 792 (71%) | 696 (67%) |  |
| *Non-Hispanic Black* | 1,563 (11%) | 418 (11%) | 421 (11%) | 367 (11%) | 357 (10%) |  |
| *Mexican American* | 1,046 (7.2%) | 215 (5.9%) | 264 (7.1%) | 250 (7.0%) | 317 (9.1%) |  |
| *Other Race - Including Multi-Racial* | 703 (7.1%) | 165 (6.6%) | 154 (7.3%) | 183 (6.7%) | 201 (8.0%) |  |
| *Other Hispanic* | 385 (4.7%) | 78 (3.6%) | 87 (4.6%) | 101 (5.0%) | 119 (5.8%) |  |
| **BMI** |  |  |  |  |  | **<0.001** |
| *Normal(<25)* | 1,882 (30%) | 453 (28%) | 462 (30%) | 453 (28%) | 514 (33%) |  |
| *Obese(≥30)* | 2,670 (39%) | 761 (45%) | 654 (37%) | 677 (40%) | 578 (33%) |  |
| *Overweight(≥25,<30)* | 2,217 (31%) | 481 (27%) | 575 (33%) | 563 (32%) | 598 (34%) |  |
| **Education** |  |  |  |  |  | **<0.001** |
| *9-11th Grade (Includes 12th grade with no diploma)* | 846 (8.5%) | 266 (10%) | 211 (8.9%) | 198 (8.0%) | 171 (6.8%) |  |
| *College Graduate or above* | 1,600 (30%) | 245 (19%) | 385 (31%) | 459 (33%) | 511 (40%) |  |
| *High School Grad/GED or Equivalent* | 1,613 (25%) | 503 (33%) | 405 (24%) | 378 (25%) | 327 (19%) |  |
| *Less Than 9th Grade* | 545 (3.8%) | 111 (3.0%) | 131 (3.8%) | 141 (4.1%) | 162 (4.6%) |  |
| *Some College or AA degree* | 2,165 (32%) | 570 (35%) | 559 (32%) | 517 (30%) | 519 (29%) |  |
| **Marital** |  |  |  |  |  | **0.002** |
| *Divorced* | 3,664 (57%) | 818 (52%) | 908 (59%) | 946 (58%) | 992 (61%) |  |
| *Living with partner* | 492 (5.1%) | 85 (3.3%) | 131 (5.6%) | 139 (6.0%) | 137 (5.9%) |  |
| *Married* | 724 (11%) | 188 (11%) | 198 (10%) | 171 (11%) | 167 (10%) |  |
| *Never married* | 221 (2.5%) | 65 (3.1%) | 46 (2.0%) | 56 (2.5%) | 54 (2.1%) |  |
| *Separated* | 1,078 (16%) | 355 (21%) | 278 (15%) | 228 (14%) | 217 (14%) |  |
| *Widowed* | 590 (8.4%) | 184 (10.0%) | 130 (7.8%) | 153 (8.4%) | 123 (7.2%) |  |
| **PIR** |  |  |  |  |  | **0.021** |
| *High(>3.49)* | 2,295 (46%) | 492 (42%) | 555 (47%) | 596 (46%) | 652 (51%) |  |
| *Low(≤1.39)* | 1,890 (19%) | 549 (21%) | 456 (18%) | 451 (18%) | 434 (16%) |  |
| *Medium(>1.39,<=3.49)* | 2,584 (35%) | 654 (37%) | 680 (35%) | 646 (37%) | 604 (33%) |  |
| **Hypertension** | 2,857 (37%) | 662 (35%) | 722 (37%) | 758 (40%) | 715 (38%) | 0.2 |
| **Hyperlipidemia** | 4,658 (67%) | 1,141 (67%) | 1,162 (70%) | 1,190 (68%) | 1,165 (64%) | 0.14 |
| **Diabetes** | 1,233 (14%) | 277 (12%) | 316 (12%) | 358 (17%) | 282 (14%) | **0.007** |
| **Depression** | 1,559 (22%) | 478 (27%) | 396 (22%) | 370 (20%) | 315 (17%) | **<0.001** |

1Mean ± SD for continuous; n (%) for categorical

2t-test adapted to complex survey samples; chi-squared test with Rao & Scott's second-order correction

Table S2. Summary of Genome-Wide Association Studies involved in our study.

| **GWAS data set** | **Sample size** | **Cases/Controls** | **Fstatistic** |
| --- | --- | --- | --- |
| Depression | 322,580 | 113,769/ 208,811 | 46.97 |
| Feeling nervous | 373,121 |  | 45.78 |
| Feeling worry | 372,869 |  | 51.92 |
| Irritable mood | 366,726 |  | 49.38 |
| Feeling lonely | 376,352 |  | 42.83 |
| Feeling tense | 371,318 |  | 50.34 |
| Feeling guilty | 373,380 |  | 48.87 |
| Feeling hurt | 372,047 |  | 49.71 |
| Feeling fed-up | 374,971 |  | 43.89 |
| Neurociticism | 380,506 |  | 42.24 |
| Feeling miserable | 376,097 |  | 43.67 |
| Worry too long after an embarrassing experience | 367,725 |  | 50.21 |
| Experiencing mood swings | 373,733 |  | 47.48 |
| Fresh fruit intake | 446,462 |  | NA |

# Table S3. Causal Association between Fruit Intake and Depression and its Related Neuroticism Traits: Results of Two-Sample Mendelian Randomization Analysis.

| **Outcome** | **SNP** | **Methods** | **OR (95% CI)** | ***P*** |
| --- | --- | --- | --- | --- |
|  |  |  |  |  |
| Depression (broad) | 39 | MR Egger | 0.96(0.82,1.13) | 0.63 |
|  |  | Weighted median | 0.94(0.88,1.01) | 0.11 |
|  |  | Inverse variance weighted | 0.92(0.87,0.96) | 5.09E-04 |
|  |  | Simple mode | 0.95(0.82,1.11) | 0.55 |
|  |  | Weighted mode | 0.95(0.86,1.05) | 0.32 |
| Feeling lonely | 36 | MR Egger | 1.18(0.76,1.83) | 0.47 |
|  |  | Weighted median | 0.83(0.72,0.95) | 0.01 |
|  |  | Inverse variance weighted | 0.82(0.74,0.9) | 2.91E-05 |
|  |  | Simple mode | 0.8(0.6,1.06) | 0.14 |
|  |  | Weighted mode | 0.83(0.65,1.06) | 0.15 |
| Feeling miserable | 33 | MR Egger | 0.79(0.51,1.22) | 0.29 |
|  |  | Weighted median | 0.80(0.70,0.91) | 9.04E-04 |
|  |  | Inverse variance weighted | 0.79(0.72,0.87) | 2.35E-06 |
|  |  | Simple mode | 0.81(0.63,1.05) | 0.13 |
|  |  | Weighted mode | 0.80(0.64,1.01) | 0.07 |
| Experiencing mood swings | 31 | MR Egger | 1.23(0.87,1.73) | 0.26 |
|  |  | Weighted median | 1.00(0.87,1.15) | 0.97 |
|  |  | Inverse variance weighted | 0.93(0.84,1.02) | 0.13 |
|  |  | Simple mode | 1.01(0.76,1.34) | 0.94 |
|  |  | Weighted mode | 1.03(0.84,1.27) | 0.77 |
| Feeling guilty | 31 | MR Egger | 0.95(0.67,1.36) | 0.8 |
|  |  | Weighted median | 0.95(0.81,1.10) | 0.49 |
|  |  | Inverse variance weighted | 0.96(0.87,1.07) | 0.48 |
|  |  | Simple mode | 0.79(0.59,1.07) | 0.13 |
|  |  | Weighted mode | 0.93(0.75,1.15) | 0.5 |
| Worry too long  after an embarrassing experience | 20 | MR Egger | 1.10(0.65,1.85) | 0.72 |
|  |  | Weighted median | 1.04(0.88,1.23) | 0.64 |
|  |  | Inverse variance weighted | 1.08(0.95,1.22) | 0.26 |
|  |  | Simple mode | 1.01(0.76,1.35) | 0.93 |
|  |  | Weighted mode | 1.02(0.78,1.33) | 0.91 |
| Feeling nervous | 37 | MR Egger | 0.92(0.53,1.60) | 0.77 |
|  |  | Weighted median | 1.05(0.90,1.24) | 0.51 |
|  |  | Inverse variance weighted | 0.94(0.80,1.11) | 0.46 |
|  |  | Simple mode | 1.34(0.75,2.39) | 0.32 |
|  |  | Weighted mode | 1.13(0.86,1.47) | 0.39 |
| Feeling worry | 22 | MR Egger | 1.15(0.83,1.61) | 0.41 |
|  |  | Weighted median | 1.07(0.91,1.27) | 0.39 |
|  |  | Inverse variance weighted | 1.09(0.97,1.22) | 0.13 |
|  |  | Simple mode | 1.00(0.71,1.41) | 0.99 |
|  |  | Weighted mode | 0.95(0.73,1.25) | 0.74 |
| Feeling tense | 26 | MR Egger | 1.12(0.80,1.56) | 0.53 |
|  |  | Weighted median | 0.99(0.84,1.15) | 0.86 |
|  |  | Inverse variance weighted | 0.95(0.85,1.05) | 0.33 |
|  |  | Simple mode | 0.98(0.74,1.30) | 0.9 |
|  |  | Weighted mode | 0.98(0.82,1.18) | 0.84 |
| Feeling fed-up | 31 | MR Egger | 0.61(0.39,0.95) | 0.04 |
|  |  | Weighted median | 0.75(0.64,0.86) | 1.06E-04 |
|  |  | Inverse variance weighted | 0.75(0.68,0.83) | 2.78E-08 |
|  |  | Simple mode | 0.66(0.48,0.9) | 0.01 |
|  |  | Weighted mode | 0.71(0.53,0.95) | 0.03 |
| Feeling hurt | 32 | MR Egger | 1.02(0.71,1.45) | 0.93 |
|  |  | Weighted median | 0.96(0.83,1.10) | 0.55 |
|  |  | Inverse variance weighted | 0.93(0.84,1.04) | 0.20 |
|  |  | Simple mode | 1.25(0.90,1.74) | 0.19 |
|  |  | Weighted mode | 1.15(0.90,1.47) | 0.28 |
| Irritable mood | 28 | MR Egger | 0.82(0.57,1.18) | 0.29 |
|  |  | Weighted median | 0.87(0.74,1.03) | 0.12 |
|  |  | Inverse variance weighted | 0.89(0.79,0.99) | 0.03 |
|  |  | Simple mode | 1.21(0.87,1.69) | 0.28 |
|  |  | Weighted mode | 0.82(0.65,1.02) | 0.08 |
| Neurociticism | 25 | MR Egger | 0.66(0.38,1.13) | 0.14 |
|  |  | Weighted median | 0.84(0.72,0.99) | 0.04 |
|  |  | Inverse variance weighted | 0.85(0.76,0.96) | 0.01 |
|  |  | Simple mode | 0.82(0.59,1.15) | 0.27 |
|  |  | Weighted mode | 0.81(0.61,1.06) | 0.14 |

# Table S4. Causal Association between Depression and its Related Neuroticism Traits and Fruit Intake: Results of Reverse Mendelian Randomization Analysis.

| **Exposure** | **SNP** | **Methods** | **OR (95% CI)** | ***P*** |
| --- | --- | --- | --- | --- |
|  |  |  |  |  |
| Depression (broad) | 7 | MR Egger | 1.27(0.8,2.03) | 0.36 |
|  |  | Weighted median | 1.03(0.87,1.21) | 0.76 |
|  |  | Inverse variance weighted | 1(0.88,1.13) | 0.97 |
|  |  | Simple mode | 1.1(0.86,1.4) | 0.48 |
|  |  | Weighted mode | 1.06(0.84,1.34) | 0.65 |
| Feeling lonely | 5 | MR Egger | 1.01(0.4,2.54) | 0.98 |
|  |  | Weighted median | 0.87(0.77,0.98) | 0.02 |
|  |  | Inverse variance weighted | 0.85(0.74,0.97) | 0.02 |
|  |  | Simple mode | 0.81(0.66,0.99) | 0.11 |
|  |  | Weighted mode | 0.86(0.71,1.04) | 0.19 |
| Feeling miserable | 10 | MR Egger | 1.03(0.86,1.23) | 0.76 |
|  |  | Weighted median | 0.99(0.92,1.07) | 0.76 |
|  |  | Inverse variance weighted | 0.98(0.93,1.04) | 0.52 |
|  |  | Simple mode | 1.03(0.91,1.17) | 0.64 |
|  |  | Weighted mode | 1.03(0.91,1.17) | 0.64 |
| Experiencing mood swings | 15 | MR Egger | 0.71(0.54,0.94) | 0.03 |
|  |  | Weighted median | 0.9(0.84,0.96) | 8.44E-04 |
|  |  | Inverse variance weighted | 0.9(0.86,0.94) | 2.05E-05 |
|  |  | Simple mode | 0.83(0.72,0.96) | 0.02 |
|  |  | Weighted mode | 0.83(0.72,0.96) | 0.03 |
| Feeling guilty | 4 | MR Egger | 0.43(0.08,2.48) | 0.45 |
|  |  | Weighted median | 0.9(0.82,1) | 0.04 |
|  |  | Inverse variance weighted | 0.93(0.85,1) | 0.06 |
|  |  | Simple mode | 0.9(0.79,1.03) | 0.21 |
|  |  | Weighted mode | 0.9(0.79,1.03) | 0.22 |
| Worry too long  after an embarrassing experience | 12 | MR Egger | 1.18(0.92,1.5) | 0.22 |
|  |  | Weighted median | 0.99(0.92,1.05) | 0.66 |
|  |  | Inverse variance weighted | 1(0.95,1.05) | 0.94 |
|  |  | Simple mode | 0.97(0.87,1.08) | 0.59 |
|  |  | Weighted mode | 0.96(0.87,1.05) | 0.41 |
| Feeling nervous | 9 | MR Egger | 0.83(0.65,1.06) | 0.17 |
|  |  | Weighted median | 1.03(0.95,1.12) | 0.42 |
|  |  | Inverse variance weighted | 0.99(0.93,1.04) | 0.62 |
|  |  | Simple mode | 1.06(0.92,1.22) | 0.46 |
|  |  | Weighted mode | 1.06(0.92,1.22) | 0.46 |
| Feeling worry | 23 | MR Egger | 0.96(0.65,1.40) | 0.82 |
|  |  | Weighted median | 1.03(0.97,1.09) | 0.35 |
|  |  | Inverse variance weighted | 1.01(0.95,1.08) | 0.73 |
|  |  | Simple mode | 1.06(0.93,1.21) | 0.36 |
|  |  | Weighted mode | 1.06(0.94,1.20) | 0.36 |
| Feeling tense | 10 | MR Egger | 0.83(0.53,1.3) | 0.45 |
|  |  | Weighted median | 1(0.94,1.08) | 0.89 |
|  |  | Inverse variance weighted | 0.98(0.93,1.04) | 0.56 |
|  |  | Simple mode | 1.02(0.92,1.13) | 0.73 |
|  |  | Weighted mode | 1.02(0.91,1.14) | 0.74 |
| Feeling fed-up | 19 | MR Egger | 1.17(0.90,1.53) | 0.26 |
|  |  | Weighted median | 0.92(0.87,0.98) | 0.01 |
|  |  | Inverse variance weighted | 0.95(0.89,1.01) | 0.08 |
|  |  | Simple mode | 0.87(0.77,0.98) | 0.04 |
|  |  | Weighted mode | 0.88(0.77,0.99) | 0.05 |
| Feeling hurt | 13 | MR Egger | 1.12(0.75,1.67) | 0.60 |
|  |  | Weighted median | 1.01(0.94,1.08) | 0.89 |
|  |  | Inverse variance weighted | 0.98(0.94,1.04) | 0.54 |
|  |  | Simple mode | 1.03(0.91,1.16) | 0.65 |
|  |  | Weighted mode | 1.03(0.91,1.16) | 0.66 |
| Irritable mood | 12 | MR Egger | 0.83(0.6,1.16) | 0.31 |
|  |  | Weighted median | 1.01(0.95,1.08) | 0.72 |
|  |  | Inverse variance weighted | 1.02(0.97,1.07) | 0.40 |
|  |  | Simple mode | 1.02(0.91,1.15) | 0.73 |
|  |  | Weighted mode | 1.02(0.92,1.12) | 0.73 |
| Neurociticism | 39 | MR Egger | 1.09(0.92,1.29) | 0.35 |
|  |  | Weighted median | 1(0.96,1.04) | 0.89 |
|  |  | Inverse variance weighted | 0.98(0.96,1.01) | 0.16 |
|  |  | Simple mode | 1.01(0.93,1.1) | 0.77 |
|  |  | Weighted mode | 1.01(0.94,1.09) | 0.74 |

# Figure S1. Visualized leave-one-out result graph.


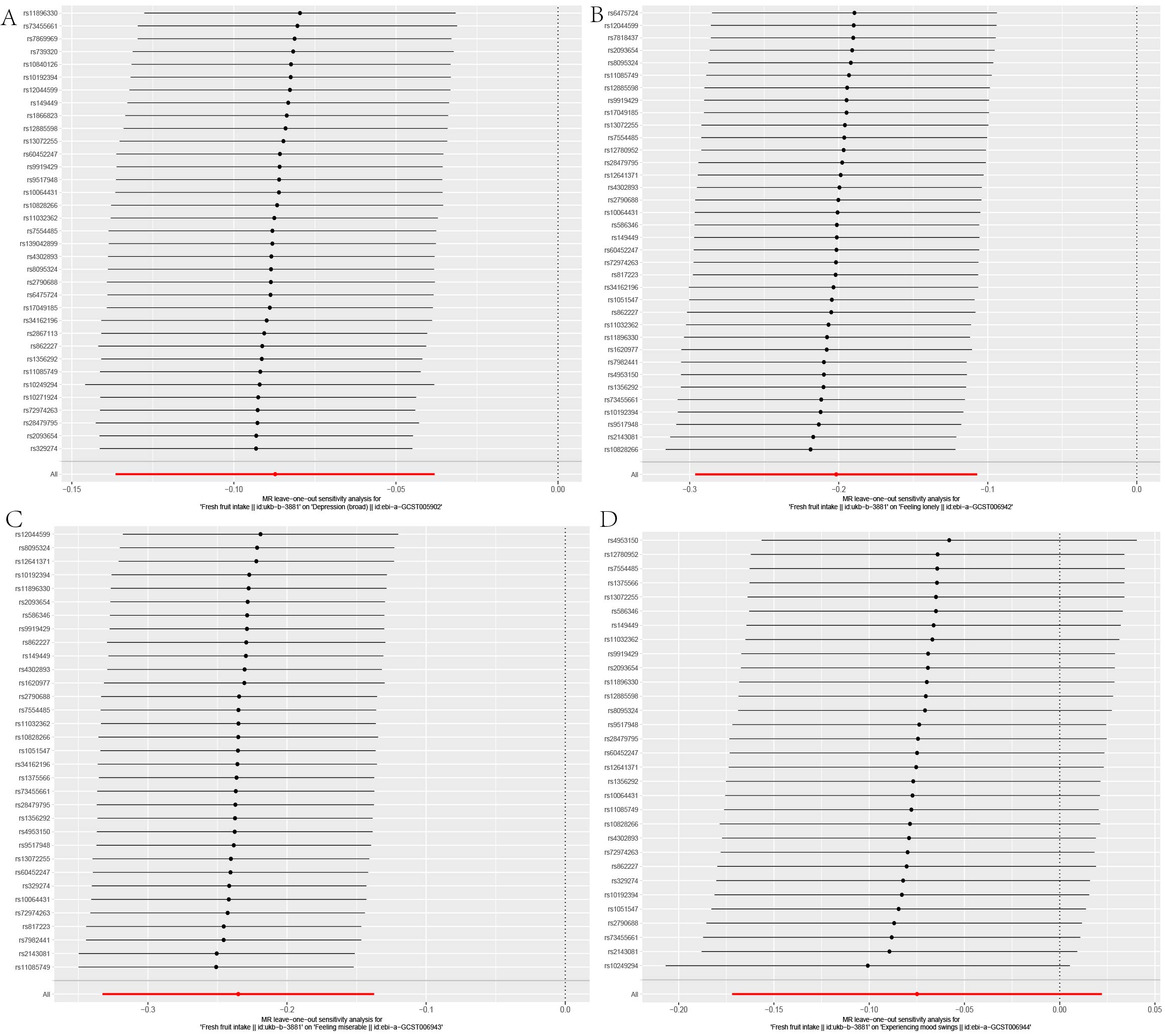


The leave-one analysis in the two-sample Mendelian randomization analysis showed that no SNP had a significant effect on the outcome (all rows were on the same side of 0). The exposures were all fruit intake, and the outcomes were as follows: (A) Depression (broad); (B) Feeling lonely; (C) Feeling miserable; (D) Experiencing mood swings

# Figure S2. Visualized leave-one-out result graph.


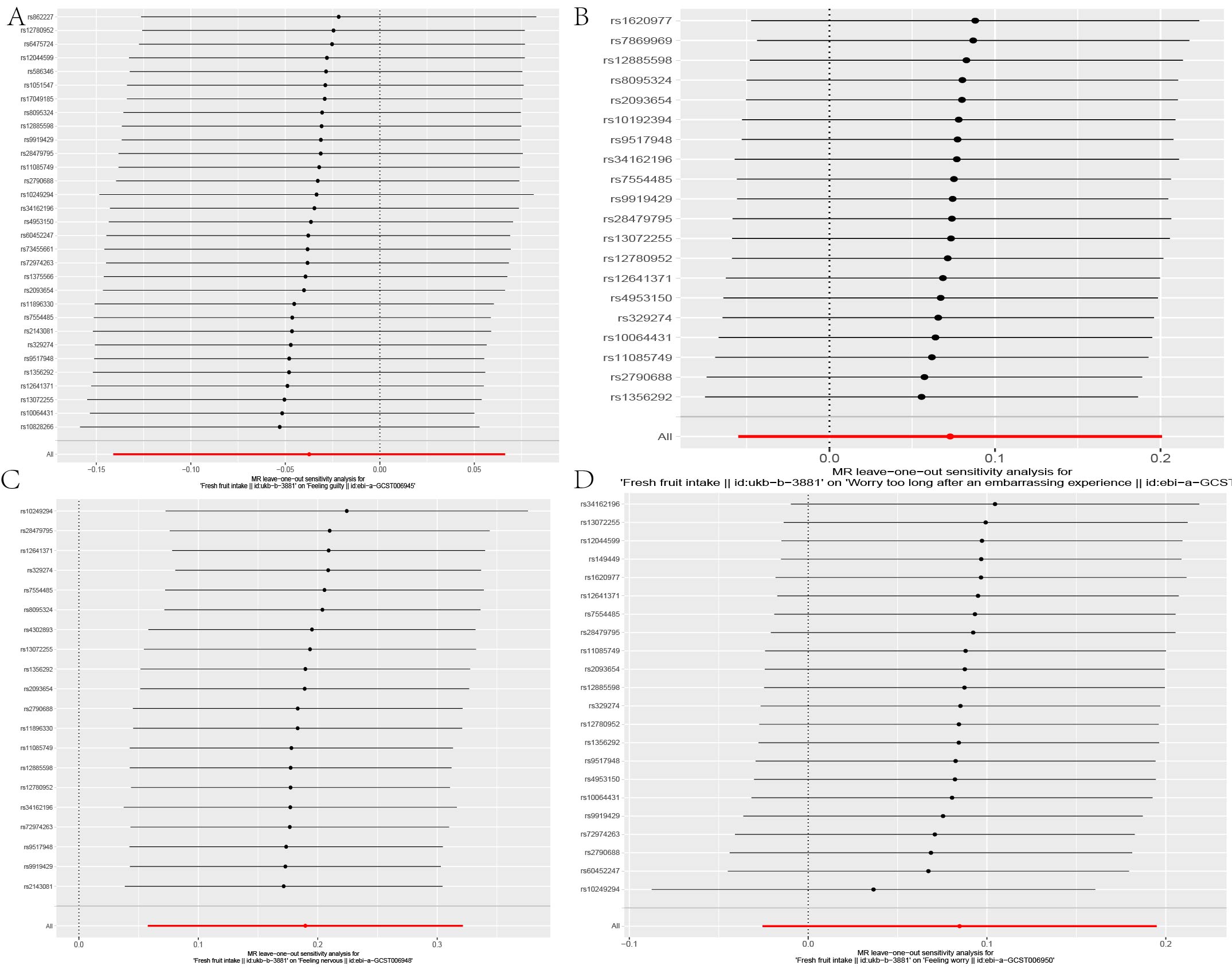


The leave-one analysis in the two-sample Mendelian randomization analysis showed that no SNP had a significant effect on the outcome (all rows were on the same side of 0). The exposure were all fruit intake, and the outcomes were as follows: (A) Feeling guilty; (B) Worry too long after an embarrassing experience; (C) Feeling nervous; (D) Feeling worry

# Figure S3. Visualized leave-one-out result graph.


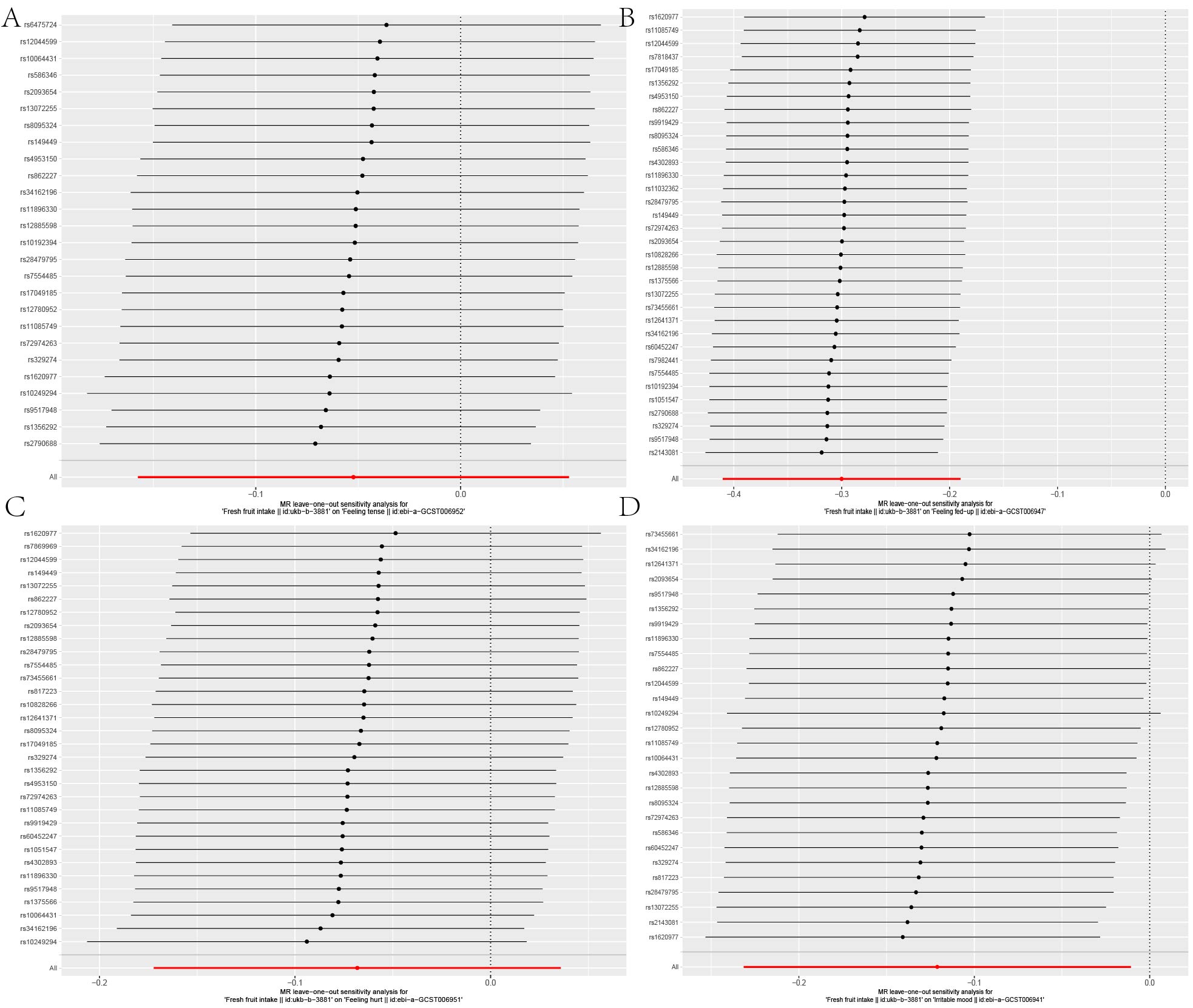


The leave-one analysis in the two-sample Mendelian randomization analysis showed that no SNP had a significant effect on the outcome (all rows were on the same side of 0). The exposure were all fruit intake, and the outcomes were as follows: (A) Feeling tense; (B) Feeling fed-up; (C) Feeling hurt; (D) Irritable mood

# Figure S4. Visualized leave-one-out result graph.


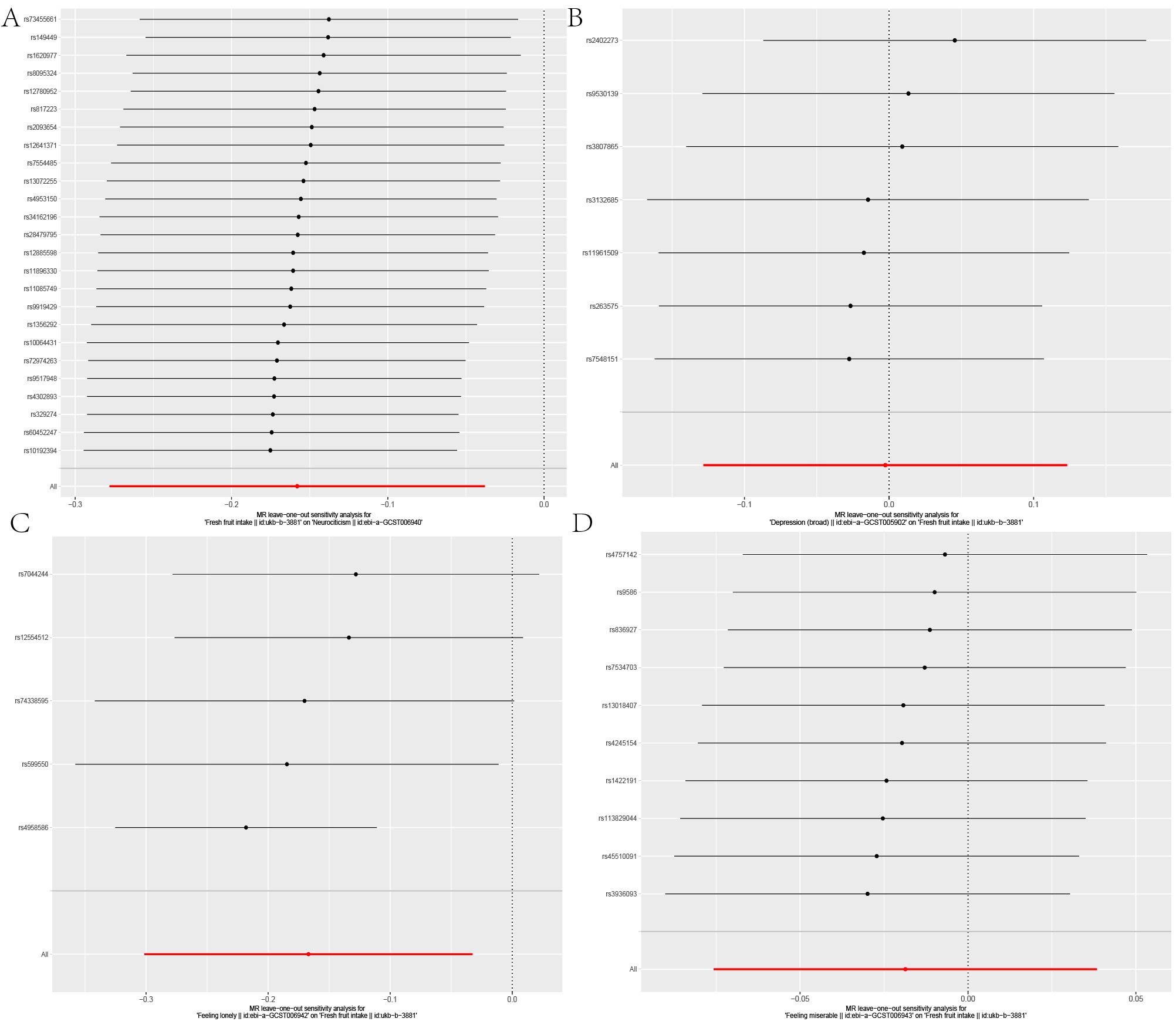


The leave-one analysis in the two-sample Mendelian randomization analysis showed that no SNP had a significant effect on the outcome (all rows were on the same side of 0). (A) The exposure was fruit intake, and the outcome was neuroticism (B) exposure: depression (broad), outcome: fruit intake (C) exposure: feeling lonely, outcome: fruit intake; (D) exposure: feeling miserable, outcome: fruit intake

# Table S5. Causal association and sensitivity analysis between Depression and its Related Neuroticism Traits and Fruit Intake in Reverse Mendelian Randomization analysis.

| **Exposure** | **Outcome** | **Cochran Q- P.value** | **MR-Egger Intercept-P.value** | **MR-PRESSO- P.value** |
| --- | --- | --- | --- | --- |
| Depression | Fruit intake | 0.40 | 0.34 | 0.44 |
| Feeling nervous |  | 0.45 | 0.19 | 0.47 |
| Feeling worry |  | 0.82 | 0.33 | 0.82 |
| Irritable mood |  | 0.25 | 0.42 | 0.26 |
| Feeling lonely |  | 0.02 | 0.73 | 0.06 |
| Feeling tense |  | 0.43 | 0.89 | 0.45 |
| Feeling guilty |  | 0.61 | 0.48 | 0.65 |
| Feeling hurt |  | 0.60 | 0.55 | 0.59 |
| Feeling fed-up |  | 0.79 | 0.59 | 0.79 |
| Neurociticism |  | 0.19 | 0.31 | 0.21 |
| Feeling miserable |  | 0.79 | 0.59 | 0.79 |
| Worry too long after an embarrassing experience |  | 0.81 | 0.23 | 0.81 |
| Experiencing mood swings |  | 0.22 | 0.11 | 0.25 |

The MR-Egger Intercept and MR-PRESSO P-values in the table are used to investigate the presence of horizontal pleiotropy. A P-value > 0.05 indicates the absence of horizontal pleiotropy, suggesting that the study aligns with the basic assumptions of Mendelian randomization. On the other hand, the P-value of Cochran's Q test explores the presence of heterogeneity. A P-value > 0.05 indicates no significant heterogeneity, indicating an association between the instrumental variables and phenotype.

# Figure S5. Visualized leave-one-out result graph.


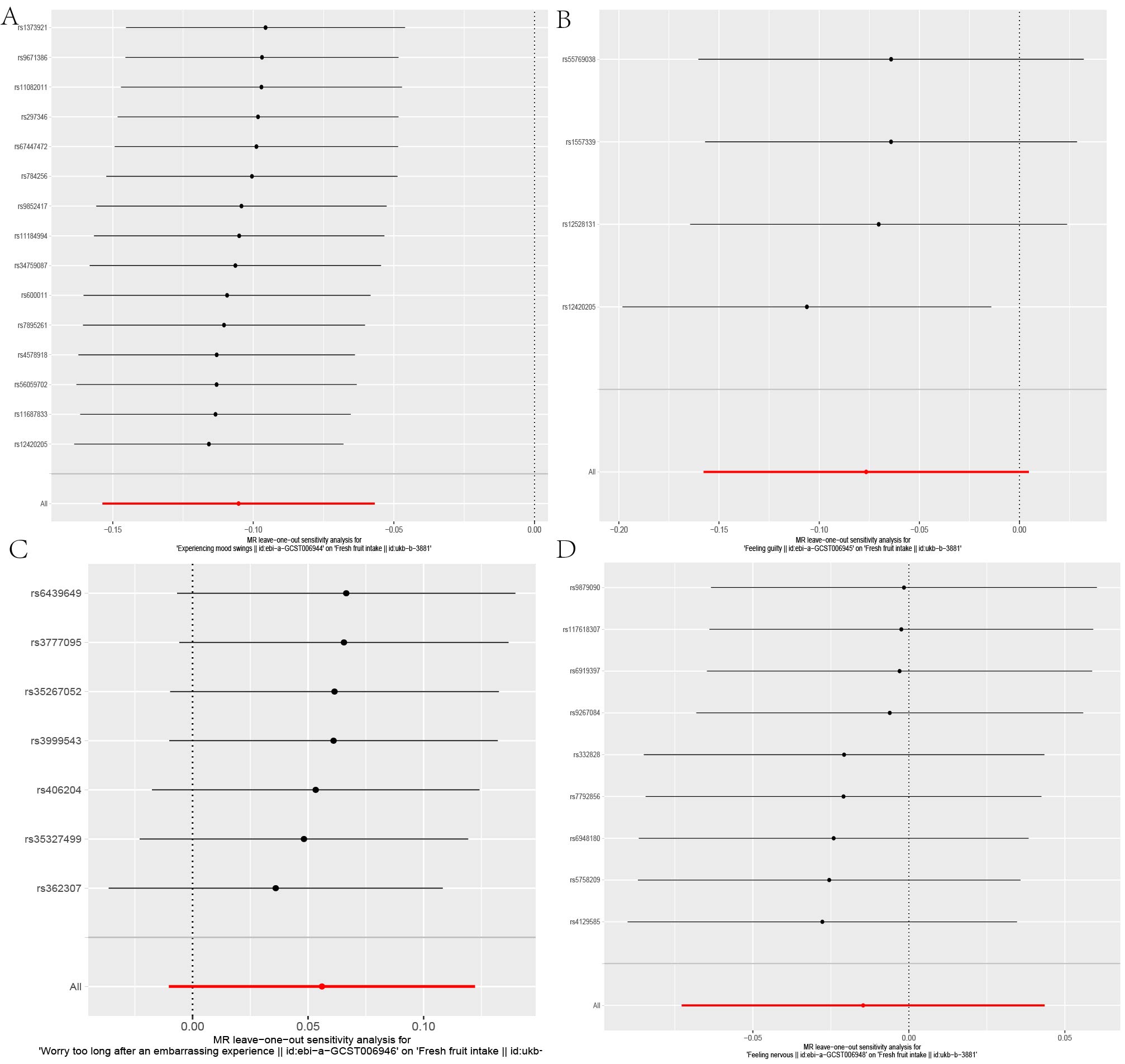


The leave-one analysis in the two-sample Mendelian randomization analysis showed that no SNP had a significant effect on the outcome (all rows were on the same side of 0). The outcome were all fruit intake, and the exposures were as follows: (A) Experiencing mood swings; (B) Feeling guilty; (C) Worry too long after an embarrassing experience; (D) Feeling nervous

# Figure S6. Visualized leave-one-out result graph.


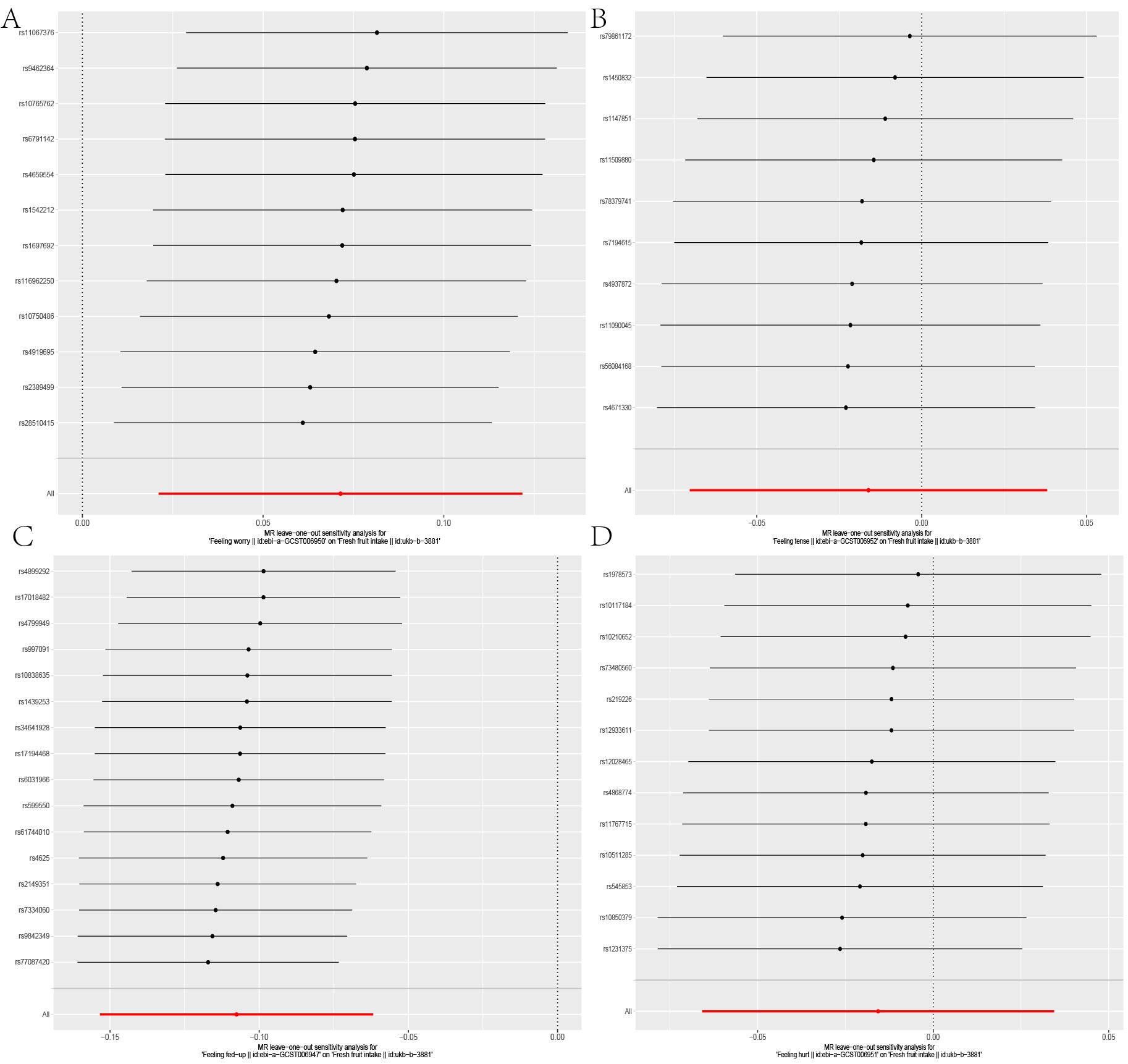


The leave-one analysis in the two-sample Mendelian randomization analysis showed that no SNP had a significant effect on the outcome (all rows were on the same side of 0). The outcome were all fruit intake, and the exposures were as follows: (A) Feeling worry; (B) Feeling tense; (C) Feeling fed-up; (D) Feeling hurt

# Figure S7. Visualized leave-one-out result graph.


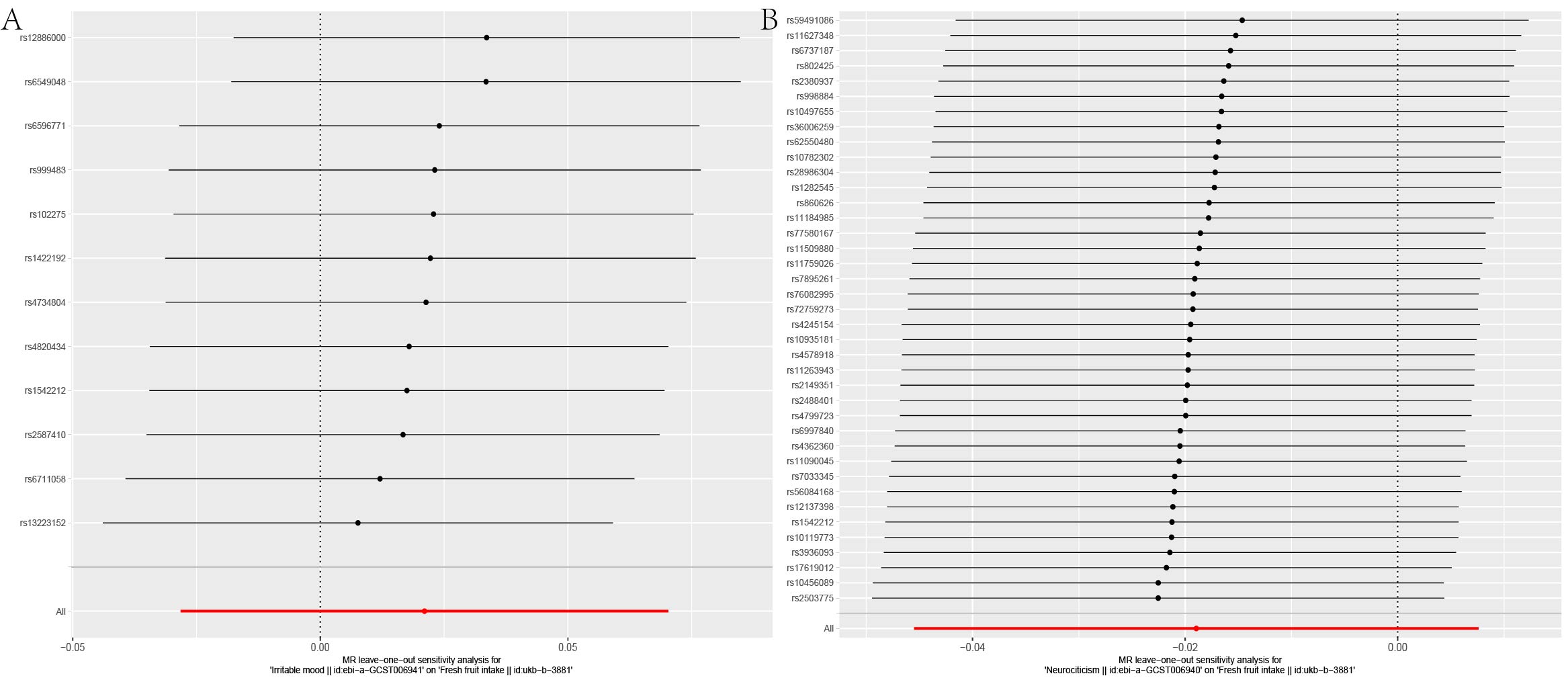


The leave-one analysis in the two-sample Mendelian randomization analysis showed that no SNP had a significant effect on the outcome (all rows were on the same side of 0). The outcome were all fruit intake, and the exposures were as follows: (A) Irritable mood; (B) Neurociticism.

# Table S6. Specific Values of Daily Fruit Intake (>1.45 cups) across Various Fruit Types Based on FPED Fruit Nutrient Database.

| **Fruit type** | **Weight of one cup**  **Equivalent (g)** | **Recommended intake (g)** |
| --- | --- | --- |
| All types of berries, raw | 145 | 210.25 |
| All types of berries, frozen, sweetened | 165 | 239.25 |
| Citrus fruit, oranges | 185 | 268.25 |
| All melon types (except, watermelon), raw | 170 | 246.5 |
| Watermelon | 150 | 217.5 |
| Fruit juice (100%) | 250 | 362.5 |
| Fruit juice concentrate, frozen | 70 | 101.5 |
| Canned fruit in juice pack(65% fruit assumed, 35% juice) | 245 | 355.25 |
| Canned fruit in water pack(65% fruit assumed) | 245 | 355.25 |
| Canned fruit in light syrup(65% fruit assumed; added sugars computed) | 250 | 362.5 |
| Canned fruit in heavy pack(65% fruit assumed; added sugars computed) | 255 | 369.75 |
| Canned fruit water or juice pack, drained | 190 | 275.5 |
| Canned fruit syrup pack, drained | 200 | 290 |
| Applesauce | 245 | 355.25 |
| Fruit nectar(40% fruit juice assumed; added sugars computed) | 250 | 362.5 |
| Fruit juice drinks(15% fruit juice assumed; added sugars computed) | 250 | 362.5 |
